# SOCS2 is part of a highly prognostic 4-gene signature in AML and promotes disease aggressiveness

**DOI:** 10.1038/s41598-019-45579-0

**Published:** 2019-06-24

**Authors:** Chi Huu Nguyen, Tobias Glüxam, Angela Schlerka, Katharina Bauer, Alexander M. Grandits, Hubert Hackl, Oliver Dovey, Sabine Zöchbauer-Müller, Jonathan L. Cooper, George S. Vassiliou, Dagmar Stoiber, Rotraud Wieser, Gerwin Heller

**Affiliations:** 1 Department of Medicine I, Division of Oncology, Medical University of Vienna, Vienna, Austria; 2 Comprehensive Cancer Center, Vienna, Austria; 3 Institute of Science and Technology Austria, Vienna, Austria; 4 Division of Bioinformatics, Biocenter, Medical University of Innsbruck, Innsbruck, Austria; 5 Wellcome Trust Sanger Institute, Wellcome Trust Genome Campus, Hinxton, UK; 6 Ludwig Boltzmann Institute for Cancer Research, Vienna, Austria; 7 Institute of Pharmacology, Center for Physiology and Pharmacology, Medical University of Vienna, Vienna, Austria; 8 Institute of Pharmacology and Toxicology, Department for Biomedical Sciences, University of Veterinary Medicine Vienna, Vienna, Austria

**Keywords:** Acute myeloid leukaemia, Cancer stem cells

## Abstract

Acute myeloid leukemia (AML) is a heterogeneous disease with respect to its genetic and molecular basis and to patients´ outcome. Clinical, cytogenetic, and mutational data are used to classify patients into risk groups with different survival, however, within-group heterogeneity is still an issue. Here, we used a robust likelihood-based survival modeling approach and publicly available gene expression data to identify a minimal number of genes whose combined expression values were prognostic of overall survival. The resulting gene expression signature (4-GES) consisted of 4 genes (*SOCS2*, *IL2RA*, *NPDC1*, *PHGDH*), predicted patient survival as an independent prognostic parameter in several cohorts of AML patients (total, 1272 patients), and further refined prognostication based on the European Leukemia Net classification. An oncogenic role of the top scoring gene in this signature, *SOCS2*, was investigated using *MLL-AF9* and *Flt3*-ITD/*NPM1c* driven mouse models of AML. *SOCS2* promoted leukemogenesis as well as the abundance, quiescence, and activity of AML stem cells. Overall, the 4-GES represents a highly discriminating prognostic parameter in AML, whose clinical applicability is greatly enhanced by its small number of genes. The newly established role of *SOCS2* in leukemia aggressiveness and stemness raises the possibility that the signature might even be exploitable therapeutically.

## Introduction

Acute myeloid leukemia (AML) is a highly heterogeneous disease in terms of clinical course and outcome: some patients do not tolerate, or are refractory to induction chemotherapy, many achieve a remission but go on to relapse with frequently therapy resistant disease, while yet others attain a permanent remission and long-term survival. AML is organized in a hierarchic manner: leukemic stem cells (LSCs) at the apex of this hierarchy give rise to the bulk of the leukemic cells (LCs), exhibit higher resistance to chemotherapy than the latter, and represent the cellular source of relapse^1^. The clinical heterogeneity of AML is related to a number of parameters, of which patient age as well as genetic and molecular alterations present in the malignant cells are of particular importance^2–5^. Numerous recurrent chromosome aberrations and point mutations have been identified in AML, and shown to act as drivers of the disease as well as to have prognostic significance^2–6^. In addition, AML is associated with extensive alterations of the transcriptome, and, like genetic aberrations, changes in gene expression contribute to leukemogenesis and may represent therapeutic targets^3,7–10^. With respect to prognostication, transcriptional patterns may even outperform the clinical and genetic parameters commonly used for this purpose^11^. Accordingly, a number of studies have identified gene expression signatures that were associated with survival, and in several cases could be cross-validated in additional data sets and even used to refine the European Leukemia Net (ELN) score^7,12–20^, which integrates cytogenetic and mutational information and is the current gold standard for prognostication in AML^21^. However, some of these signatures were established by applying a prior gene selection process that may impede their general applicability, and most contain too many genes to be realistically implemented in clinical routine.

*Suppressor Of Cytokine Signalling 2* (*SOCS2*) is transcriptionally activated by JAK-STAT signalling and encodes a negative feedback regulator of this pathway, which is aberrantly activated in many cancers including AML^22^. *SOCS2* would therefore be predicted to act as a tumor suppressor, and was indeed down-regulated in several types of solid tumors^22^. On the other hand, oncogenic roles of *SOCS2* were reported, e.g., in colon and prostate cancer^23–25^. In the healthy murine hematopoietic system, *Socs2* was expressed at high levels in stem cells (HSCs) and down-regulated during differentiation^26,27^. A requirement for *Socs2* function in HSCs, however, became apparent only in specific experimental settings like 5-Fluorouracil treatment or a longer series of consecutive transplantations^26,27^. As for hematopoietic malignancies, *SOCS2* expression was significantly increased in patients with chronic myeloid leukemia (CML) in blast crisis as compared to chronic phase patients and healthy controls^28^, but its absence did not alter the latency or histopathologic features of CML like disease in mice transplanted with *BCR-ABL1* transduced bone marrow cells^26^. A crucial role of JAK-STAT signalling in AML, including in LSCs, is well documented^29^, but so far, little is known about the role of *SOCS2* in this disease.

Here, we report the establishment of a gene expression signature that was composed of 4 genes and consistently associated with survival in 7 cohorts of AML patients with publicly available gene expression and survival data. The top gene in this signature was *SOCS2*. Experiments using mouse models of AML as well as malignant human myeloid cell lines demonstrated a role of *SOCS2* in disease aggressiveness and stemness.

## Results

### Establishment of a 4-gene expression signature with prognostic value in AML

**Figure 1:**
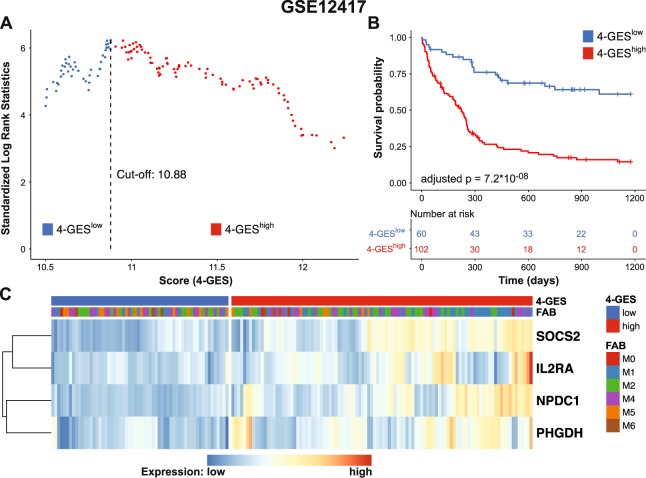
Prognostic value of the 4-GES in 162 cytogenetically normal AML patients (GSE12417, cohort 1). (**A**) Cut-off value for stratification of AML patients into 4-GES^low^ (blue) and 4-GES^high^ (red) was calculated by maximally selected rank statistics. (**B**) Kaplan Meier curves for overall survival of 162 AML patients classified as 4-GES^low^ or 4-GES^high^. Log rank tests were calculated and p-values were adjusted for multiple testing according to Altman *et al*.^58^. **(C)** Heatmap summarizing expression values of *SOCS2*, *IL2RA*, *NPDC1* and *PHGDH* in 4-GES^low^ (blue) and 4-GES^high^ (red) AML patients. Blue, low expression; red, high expression.

Cohort 1 of data set GSE12417 was used as training set, because it includes patients of all age groups, but is restricted to AML with a normal karyotype, which is the prognostically most heterogeneous of the cytogenetically defined subgroups of AML (Table 1)^13^. After removal of an MDS sample, gene expression data of 162 cytogenetically normal AML patients remained for model calculation. A forward gene selection was employed and the optimal prognostic model was selected by using the criterion of minimal AIC, an approach to minimize model complexity while maintaining maximum fit of the model to the data (Table 2). This approach resulted in the identification of 4 genes (*SOCS2*, *IL2RA*, *NPDC1* and *PHGDH*), whose combined expression values were prognostic of OS in this cohort of patients. A 4-gene expression score (4-GES) was calculated using the expression values and the β-coefficients from a multivariable Cox regression analysis of each of the 4 genes. Based on the resulting score, patients were classified into low risk (N = 60) and high risk (N = 102) groups using maximally selected rank statistics (Fig. 1A). Kaplan Meier analysis and log rank testing revealed that AML patients with a high 4-GES had significantly shorter OS than patients with a low 4-GES (median survival: 223 days vs. not reached, adjusted p = 7.2 * 10^−08^; Fig. 1B). GSE12417 data were generated using Affymetrix arrays, and on these, many genes are represented by more than one probe set. The forward selection process used to establish the 4-GES picked only one of these per gene. However, all other probe sets for the 4 genes were also prognostic of OS when analysed individually (adjusted p < 0.05 in all cases; Supplementary Fig. S1).

**Table 1:** Summary of AML gene expression data sets retrieved from GEO and TCGA/Cancer Browser databases.

Accession number	Platform	Patients (N)^a^	Age^b^	Cytogenetics
GSE12417, cohort 1	Affymetrix, HG-U133A	162	58 (17–83)	Normal
GSE12417, cohort 2	Affymetrix, HG-U133_Plus_2	78	62 (18–85)	Normal
GSE6891, cohort 1	Affymetrix, HG-U133_Plus_2	222	43 (15–60)	Heterogeneous
GSE6891, cohort 2	Affymetrix, HG-U133_Plus_2	185	45 (17–60)	Heterogeneous
GSE37642	Affymetrix, HG-U133A	379	57 (18–83)	Heterogeneous
GSE71014	Illumina HumanHT-12 V4.0	104	na	Normal
TCGA_LAML	RNA-sequencing	142	60 (18–88)	Heterogeneous

^a^Number of patients after exclusion of samples with FAB M3/unknown FAB type or with MDS. ^b^Age in years, median (range). na, not available.

**Table 2:** Survival associated gene expression model identified by forward gene selection using GSE12417 cohort 1.

Probe ID	Gene Symbol	nloglik	AIC	Selected
203373_at	*SOCS2*	452.01	910.02	*
218086_at	*NPDC1*	449.26	906.52	*
211269_s_at	*IL2RA*	447.91	905.81	*
201397_at	*PHGDH*	444.23	900.46	*
218966_at	*MYO5C*	444.23	902.45	
209386_at	*TM4SF1*	443.2	902.39	
203372_s_at	*SOCS2*	443.17	904.33	
201540_at	*FHL1*	441.85	903.71	
211597_s_at	*HOPX*	440.17	902.33	

Nloglik, negative log likelihood; AIC, Akaike information criterion; AIC is provided for the prognostic model including the respective gene and all genes above that gene in the list.

In a multivariable setting, the 4-GES remained significantly associated with OS after adjusting for patient age (p = 8.8 * 10^−08^, HR = 3.8; Table 3). The expression pattern of *SOCS2*, *IL2RA*, *NPDC1*, and *PHGDH* in GSE12417 cohort 1 is shown in Fig. 1C. Application of the 4-GES to GSE12417 cohort 2, which contains samples from 78 patients with AML, yielded similar results as described for cohort 1 (p = 0.035, HR = 4.58 after adjusting for age; Table 3). Overall, these findings demonstrate that high expression of *SOCS2*, *IL2RA*, *NPDC1* and *PHGDH* may be of prognostic relevance for AML patients. Thus, we proceeded to validate the model in 5 additional patient cohorts.

**Table 3:** Multivariable Cox regression analysis for overall survival of AML patients.

Data set/cohort	Variable	4-GES			L-24			M-7			W-3		
		HR	95% CI	p-value	HR	95% CI	p-value	HR	95% CI	p-value	HR	95% CI	p-value
**GSE12417/1**	GE score, high *vs*. low	**3.8**	**2.3–6.2**	**8.8** * **10**^**−08**^	**2.25**	**1.5–3.4**	**7.5** * **10**^**−05**^	na	Na	na	**1.51**	**1.07–2.14**	**0.021**
	Age (years)	**1.02**	**1.0–1.0**	**0.006**	**1.03**	**1.0–1.04**	**0.0003**	na	Na	na	**1.03**	**1.01–1.04**	**0.0004**
**GSE12417/2**	GE score, high *vs*. low	**4.58**	**1.1–18.9**	**0.035**	ns	ns	ns	**2.68**	**1.5–4.9**	**0.0016**	**2.01**	**1.1–3.7**	**0.022**
	Age (years)	**1.04**	**1.0–1.1**	**0.008**	ns	ns	ns	**1.03**	**1.0–1.06**	**0.0063**	**1.03**	**1.0–1.1**	**0.0079**
**GSE6891/1**	GE score, high *vs*. low	**1.69**	**1.2–2.5**	**0.008**	1.1	0.8–1.6	0.56	**2.0**	**1.4–2.8**	**5.3** * **10**^**−05**^	ns	ns	ns
	Cytogenetic risk^a^	**1.91**	**1.5–2.5**	**1.4** * **10**^**−06**^	**1.97**	**1.5–2.6**	**3.7** * **10**^**−07**^	**1.98**	**1.6–2.5**	**2.9** * **10**^**−08**^	ns	ns	ns
	CEBPAm (w/m/b)	0.79	0.1–6.4	0.828	0.84	0.1–8.1	0.878	0.95	0.1–6.4	0.957	ns	ns	ns
	CEBPAm (yes/no)	0.94	0.0–57.1	0.976	0.71	0.0–61.2	0.878	0.66	0.01–27.5	0.83	ns	ns	ns
	*EVI1* expression (+/−)	0.82	0.5–1.5	0.511	0.95	0.5–1.7	0.854	0.89	0.5–1.6	0.681	ns	ns	ns
	FLT3-ITD	1.38	0.9–2.0	0.106	**1.69**	**1.2–2.5**	**0.006**	**1.82**	**1.3–2.6**	**0.0008**	ns	ns	ns
**GSE6891/2**	GE score, high *vs*. low	**2.24**	**1.5–3.4**	**0.0001**	1.33	0.9–2.0	0.173	**1.7**	**1.1–2.5**	**0.0086**	ns	ns	ns
	Age (years)	1.01	1.0–1.03	0.281	1.01	1.0–1.03	0.128	1.01	1.0–1.03	0.102	ns	ns	ns
	Cytogenetic risk^a^	1.13	0.8–1.6	0.528	1.22	0.9–1.75	0.27	1.35	1.0–1.92	0.092	ns	ns	ns
	*EVI1* expression (+/−)	**3.73**	**1.8–7.6**	**0.0002**	**2.95**	**1.5–6.0**	**0.0025**	**2.9**	**1.4–5.8**	**0.0028**	ns	ns	ns
**GSE37642**	GE score, high *vs*. low	**2.13**	**1.6–2.8**	**1.8** * **10**^**−07**^	**1.49**	**1.2–1.9**	**0.0016**	na	Na	na	ns	ns	ns
	Age (years)	**1.03**	**1.0–1.04**	**5.6** * **10**^**−12**^	**1.03**	**1.0–1.04**	**1.5** * **10**^**−13**^	na	Na	na	ns	ns	ns
	ELN score^b^	**1.26**	**1.1–1.4**	**7.1** * **10**^**−05**^	**1.33**	**1.2–1.5**	**4.1** * **10**^**−07**^	na	Na	na	ns	ns	ns
	FAB, M1 vs. others	1.06	0.8–1.4	0.718	0.95	0.7–1.3	0.745	na	Na	na	ns	ns	ns
	FAB, M4 vs. others	1.32	1.0–1.8	0.074	**1.45**	**1.1–2.0**	**0.017**	na	Na	na	ns	ns	ns
**TCGA_LAML**	GE score, high *vs*. low	1.52	1.0–2.3	0.051	1.3	0.8–2.1	0.249	ns	Ns	ns	**2.45**	**1.4–4.3**	**0.002**
	Age (years)	**1.03**	**1.0–1.1**	**0.00013**	**1.03**	**1.0–1.1**	**8.8** * **10**^**−05**^	ns	Ns	ns	**1.04**	**1.0–1.1**	**2.3** * **10**^**−05**^
	Cytogenetic risk^a^	1.29	0.9–1.9	0.179	1.25	0.8–1.9	0.265	ns	Ns	ns	**1.49**	**1.0–2.1**	**0.033**

Parameters provided with the respective data sets were first tested in univariable analyses (Supplementary Table S1); those that resulted as significant were included in the multivariable models, whose results are summarized in this table. 4-GES, 4-gene expression score; L-24, 24-gene expression signature by Li *et al*.^16^; M-7, 7-gene expression signature by Marcucci *et al*.^19^; W-3, 3-gene expression signature by Wilop *et al*.^20^; HR, hazard ratio; CI, confidence interval; GE score, gene expression score; CEBPAm, *CEBPA* mutation; w, wild type; m, monoallelic; b, biallelic. ^a^Assignment to cytogenetic risk groups were included in the respective GEO entries. ^b^Assignment to ELN risk groups was provided by T. Herold, University of Munich, Department of Internal Medicine III, Munich, Germany. No relevant patient data were provided in GSE71014; therefore, multivariable analyses could not be performed. Significant p-values and corresponding HRs and Cis are indicated in bold letters. na, score could not be calculated because 2 signature genes were not represented on HG-U133A microarrays; ns, no statistical significance found in univariable analyses, thus, no multivariable analyses were performed.

### Validation of the 4-GES in patients with cytogenetically heterogeneous AML

**Figure 2:**
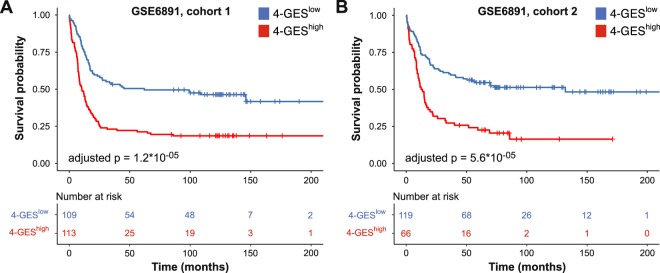
Validation of the 4-GES in gene expression data set GSE6891, containing cytogenetically heterogeneous AML patients. **(A)** Cohort 1 (N = 222), **(B)** cohort 2 (N = 185). Kaplan Meier curves for overall survival of 4-GES^low^ (blue) and 4-GES^high^ (red) patients are shown. Statistical significance was calculated using the log rank test and p-values were adjusted for multiple testing according to Altman *et al*.^58^.

To determine whether the 4-GES has prognostic value also in cytogenetically heterogeneous AML, survival analyses were performed using data set GSE6891, which consists of 2 cohorts of AML patients with variable karyotypes^30^. Because treatment of patients with AML M3, characterized by the translocation t(15;17), differs substantially from that of all other AML subgroups, the corresponding samples were removed from this and all other cytogenetically heterogeneous data sets. The 4-GES again predicted OS in a highly significant manner (adjusted p = 1.2 * 10^−05^ and adjusted p = 5.6 * 10^−05^ for cohorts 1 and 2, respectively; Fig. 2A,B). In both cohorts, the 4-GES was also significant in multivariable analyses including several other prognostic parameters as shown in Table 3. GSE6891 contains information about cytogenetic risk groups. To obtain larger groups, we merged cohorts 1 and 2 and repeated survival analyses separately for the three cytogenetic subgroups. A highly significant prognostic value of the 4-GES was seen for the cytogenetically intermediate subgroup (n = 242, adjusted p = 5.1 * 10^−06^), but not for the substantially smaller cytogenetically favourable (n = 74) and poor (n = 80) subgroups (Supplementary Fig. S2).

### The 4-GES is able to refine the ELN risk classification

**Figure 3:**
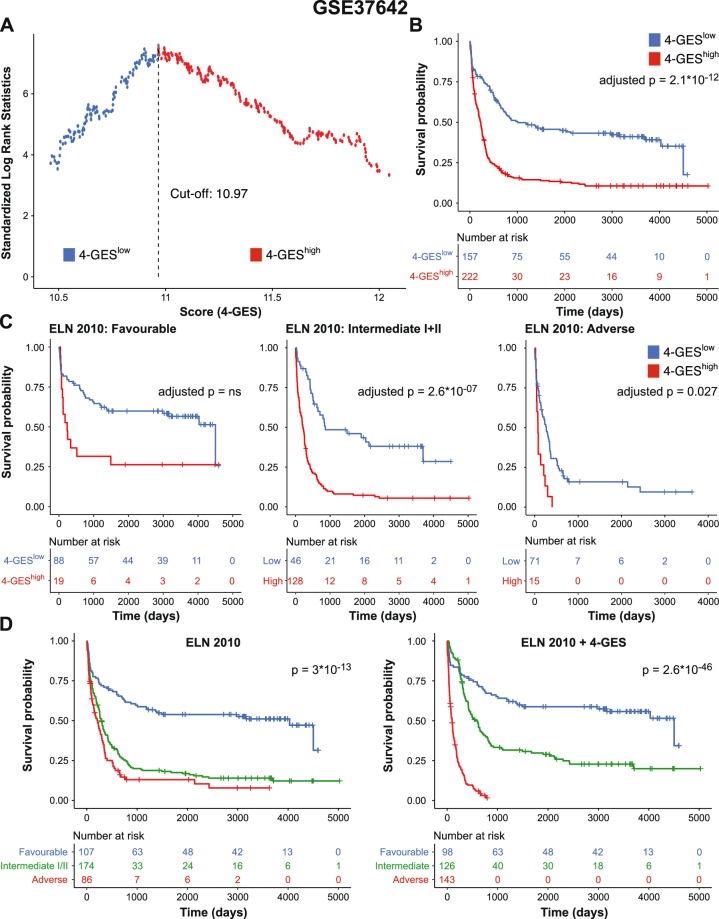
Validation of the 4-GES in gene expression data set GSE37642, consisting of 379 cytogenetically heterogeneous AML patients. **(A)** Cut-off value for stratification of AML patients into 4-GES^low^ (blue) and 4-GES^high^ (red) was calculated by maximally selected rank statistics. **(B)** Kaplan Meier curves for overall survival of 379 AML patients classified as 4-GES^low^ (blue) and 4-GES^high^ (red). **(C)** Kaplan Meier curves for overall survival of 107 ELN favourable, 174 ELN intermediate I/II and 86 ELN adverse risk AML patients stratified into 4-GES^low^ (blue) and 4-GES^high^ (red). Statistical significance was calculated using the log rank test and p-values were adjusted for multiple testing according to Altman *et al*.^58^. **(D)** Kaplan Meier curves for overall survival of AML patients stratified into favourable, intermediate, and adverse based on ELN 2010 classification (left) and ELN 2010 + 4-GES classification (right). ELN, European Leukemia Net.

Like GSE6891, GSE37642 contains data from cytogenetically heterogeneous AML^16^; in addition, ELN risk classification information was available. In a first step, the entire study population was divided into a 4-GES^high^ (N = 222) and a 4-GES^low^ (N = 157) group (Fig. 3A). Patients in the 4-GES^high^ group had significantly shorter OS than patients in the 4-GES^low^ group (adjusted p = 2.1 * 10^−12^; Fig. 3B). ELN classification was available for 367 patients: 107 were assigned to the favourable risk group, 100 to the intermediate I, 74 to the intermediate II, and 86 to the adverse risk group. Multivariable Cox regression analysis revealed that the 4-GES remained significantly associated with OS after adjusting for patient age and ELN score (adjusted p = 1.8 * 10^−07^, HR = 2.13; Table 3). Next, we investigated whether the 4-GES was prognostic also within the ELN risk groups. Stratification of the patients within the favourable, intermediate I/II, and adverse groups into 4-GES^high^ and 4-GES^low^ subgroups resulted in a clear identification of patients with shorter OS in the ELN intermediate I/II (adjusted p = 2.6 * 10^−07^) and adverse (adjusted p = 0.027) groups (Fig. 3C). Among the ELN favourable patients, the difference did not reach statistical significance after adjustment for multiple testing. To ask whether the 4-GES was able to refine the ELN classification, patients from the six groups generated in the previous analysis were combined into three new groups based on median survival: ELN favourable/4-GES^high^ patients with low median OS were re-assigned to ELN intermediate risk group, ELN intermediate/4-GES^high^ patients with low median OS were re-assigned to ELN adverse risk group, and ELN adverse/4-GES^low^ patients with high median OS were re-assigned to ELN intermediate risk group. The resulting ELN + 4-GES classification indeed substantially refined the ELN score (p = 2.6 * 10^−46^ *vs*. p = 3 * 10^−13^ for the ELN + 4-GES and the ELN scores, respectively; Fig. 3D and Supplementary Fig. S3A). Similar results were obtained when these analyses were done separately for patients younger or older than 60 years of age (Supplementary Fig. S3B).

### Validation of the 4-GES in gene expression data sets generated with alternative technologies

**Figure 4:**
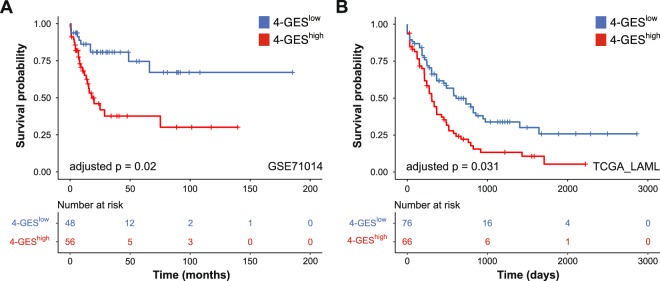
Validation of the 4-GES in the gene expression data sets **(A)** GSE71014 and **(B)** TCGA_LAML. Kaplan Meier curves for overall survival of 4-GES^low^ (blue) and 4-GES^high^ (red) patients are shown. Statistical significance was calculated using the log rank test and p-values were adjusted for multiple testing according to Altman *et al*.^58^.

To investigate whether the 4-GES retains prognostic value in AML patients whose samples were analysed on platforms other than Affymetrix microarrays, we performed survival analyses on data sets GSE71014 and TCGA_LAML^2,31^. GSE71014 consists of 104 cytogenetically normal AML samples, which were analysed using the Illumina BeadArray platform. Forty-eight patients were 4-GES^low^ and 56 were 4-GES^high^, and the latter had significantly shorter OS (adjusted p = 0.02; Fig. 4A). Multivariable analysis was not possible because no additional potentially prognostic parameters are provided in this data set.

Data set TCGA_LAML contains RNA-sequencing data from 142 AML samples (76 4-GES^low^ and 66 4-GES^high^), and a high 4-GES was significantly associated with shorter OS (adjusted p = 0.031; Fig. 4B). Multivariable analyses of TCGA_LAML, including the parameters age, cytogenetic risk score, and 4-GES, revealed a HR of 1.52 for the 4-GES, however, statistical significance was just not reached (p = 0.051, Table 3).

Overall, these findings demonstrate that the 4-GES is a highly reliable independent prognostic factor in AML.

### Comparison of the 4-GES to other prognostic gene expression signatures

As outlined above, several gene expression based scores have been proposed for the prognostication of AML. To further validate the 4-GES, we therefore compared it to a selected subset of these signatures: the 24-gene signature reported by Li *et al*.^16^ (L-24), the 7-gene signature of Marcucci *et al*.^19^ (M-7), and Wilop’s 3-gene signature^20^ (W-3). The L-24 was chosen because it was the first signature that was rigorously validated in several additional data sets and shown to be able to refine the ELN score^16^. The other two signatures were selected because, like the 4-GES, they contain <10 genes, making them particularly attractive for potential clinical use. In addition, the L-24 and the M-7 were included in a recent comparison of several prognostic scores^32^ and yielded favourable results. The L-24 and the M-7 divide patients into two prognostic groups, while the W-3 is based on three groups. There is no overlap of genes between these signatures and the 4-GES (Supplementary Table S3). In univariable analyses, the 4-GES was significant in all seven patient cohorts (Supplementary Table S4). The L-24 reached significance in 5/7 cohorts, and the M-7 in 4/5 cohorts (in the remaining two cohorts, it was not applicable because not all signature genes were represented on the array type used). The W-3 performed well in three cohorts, two of which were used to establish it (Supplementary Table S4). In multivariable analyses that included each signature together with all other available prognostic parameters, the 4-GES yielded higher hazard ratios than the other signatures in 4/6 data sets (GSE71014 could not be analyzed because no additional clinical data were provided with it), performed similar to the M-7 in one data set, and was superseded by the W-3 in the TCGA data set, which was part of the training set used to establish this signature (Table 3). In summary, therefore, the 4-GES compared favourably to other prognostic gene expression scores.

### *SOCS2* promotes AML aggressiveness and stemness

**Figure 5:**
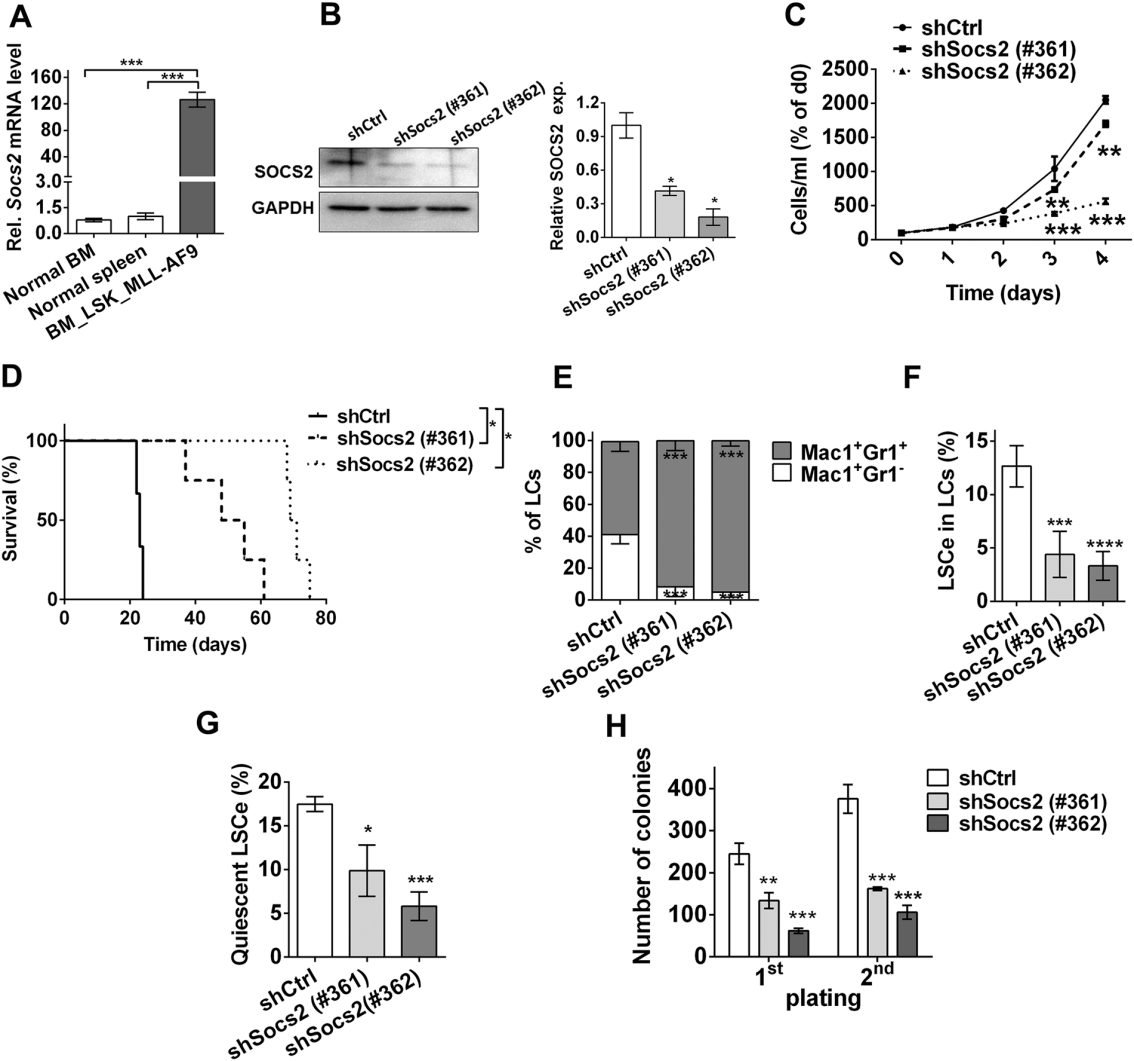
Effects of *Socs2* knock-down in an *MLL-AF9* driven mouse model of AML. **(A)** *Socs2* mRNA levels in *MLL-AF9*^+^ BM LCs and in BM and spleen cells of healthy mice were determined by qRT-PCR and normalised to those of the housekeeping gene *ß-2-microglobulin* using the ∆∆C_T_ method. Mean ± SEM of three independent experiments; ***p < 0.001 (Student’s two-tailed t-test). **(B)** Left panel, immunoblot analysis for SOCS2 expression in shCtrl or shSocs2 transduced *MLL-AF9*^+^ LCs. Right panel, quantification of immunoblot results; mean ± SEM of two independent experiments; *p < 0.05 (Student’s two-tailed t-test). **(C)** shCtrl or shSocs2 transduced *MLL-AF9*^+^ LCs were maintained in suspension culture and counted on the indicated days. Mean ± SEM of three independent experiments. **p < 0.01; ***p < 0.001 (2-way ANOVA followed by Bonferroni’s post-hoc test). **(D)** Kaplan-Meier plot of mice transplanted with shCtrl or shSocs2 transduced *MLL-AF9*^+^ LCs (300.000 Venus^+^ RFP^+^ cells per mouse, n = 4). **p < 0.01 (log-rank test). **(E**–**H)** Analyses of LCs from mice terminally ill after transplantation with shCtrl or shSocs2 transduced *MLL-AF9*^+^ LCs. Mean ± SEM of 3 independent experiments. *p < 0.05, **p < 0.01, ***p < 0.001 (2-way ANOVA followed by Bonferroni’s post-hoc test). **(E)** Myeloid differentiation of spleen LCs. Mac-1^+^ Gr-1^−^, immature LCs; Mac-1^+^ Gr-1^+^, mature LCs. **(F)** Abundance of LSC enriched cells (LSCe; Lin^−^ cKit^+^ Sca-1^−^ CD34^+^ CD16/CD32^hi^ cells) among Venus^+^ RFP^+^ spleen LCs. **(G)** Cell cycle distribution of spleen LSCe was determined by Ki67 and DAPI staining. Quiescent cells were defined as Ki67^−^ cells with a 2n DNA content. **(H)** Colony formation by BM LCs serially plated into methyl cellulose.

Because the expression of *SOCS2* was found to be most strongly associated with OS according to the robust likelihood-based approach, and expression differences between 4-GES^low^ and 4-GES^high^ AML samples were higher for *SOCS2* than for the other genes of the model, this gene was subjected to functional analysis. In normal hematopoietic cells SOCS2 levels were high in hematopoietic stem cells and substantially decreased during cell differentiation (Supplementary Fig. S4). To investigate the role of *Socs2* in leukemogenesis, a well-established mouse model of human AML driven by the fusion oncogene *MLL-AF9* was used^33^. Transplantation of Lin^−^ Sca-1^+^ c-Kit^+^ (LSK) cells transduced with pMSCV_MLL-AF9_IRES_Venus^34^ into sublethally irradiated C57BL/6 recipient mice led to a rapid-onset AML-like disease (Supplementary Fig. S5) as reported^35^. *Socs2* mRNA expression was highly up-regulated in leukemic cells (LCs) from terminally ill mice compared to normal BM or spleen cells (Fig. 5A). To knock down *Socs2*, LCs were transduced with two different shRNAs (shSocs2 #361 and #362), expressed in the lentiviral vector pLKO.1_puro_CMV_TagRFP, or with non-target shRNA (shCtrl) as a control. Venus^+^ RFP^+^ cells were isolated by flow cytometry, and down-regulation of SOCS2 by the gene specific shRNAs was confirmed by immunoblot analysis (Fig. 5B). Knock-down of *Socs2* significantly inhibited growth of primary murine LCs *in vitro* (Fig. 5C), and delayed disease onset upon transplantation into C57BL/6 recipient mice (Fig. 5D). In LCs from terminally ill mice, it effected a shift from immature (Mac-1^+^ Gr-1^−^) to more mature (Mac-1^+^ Gr-1^+^) myeloid cells (Fig. 5E). Moreover, experimental down-regulation of *Socs2* negatively affected the abundance and functional properties of LSCs. In the *MLL-AF9* model, LSCs are strongly enriched in the Lin^−^ Sca1^−^ c-Kit^+^ CD34^+^ CD16/CD32^hi^ population^35^, henceforth termed LSCe. Knock-down of *Socs2* caused a decrease of immunophenotypically defined LSCe among Venus^+^ RFP^+^ LCs (Fig. 5F), lessened the number of quiescent LSCe (Fig. 5G), and reduced clonogenicity in a serial replating assay (Fig. 5H), indicating that *Socs2* enhanced LSC abundance and function in the *MLL-AF9* mouse model of AML.

To confirm these observations, a second AML mouse model driven by the combined action of a *Flt3* gene with an activating internal tandem duplication (*Flt3*-ITD) and a mutated *nucleophosmin* gene (*NPM1c*) was employed^36^. As with the *MLL-AF9* model, spleen cells from terminally ill *Flt3*-ITD/*NPM1c* mice expressed highly elevated levels of *Socs2* mRNA compared to normal spleen cells (Supplementary Fig. S6A). Knock-down of *Socs2* in *Flt3*-ITD/*NPM1c* LCs led to a rapid and complete loss of RFP^+^ cells in culture (Supplementary Fig. S6B). While this prevented the use of these cells for transplantation experiments, it provided strong evidence for an essential role of *Socs2* in the proliferation and/or survival of *Flt3*-ITD/*NPM1c* driven LCs. Noteworthy, analyses of expression microarray data from sorafenib or DMSO treated murine pro-B cells with stable FLT3-ITD expression revealed significantly lower expression of *Socs2*, *Il2ra* and *Phgdh* in sorafenib treated cells compared to DMSO controls (Supplementary Fig. S7). Further confirming the role of *SOCS2* in leukemic cell proliferation, retroviral expression of this gene in the malignant human myeloid cell lines U937 and HL60 caused significant increases in cell numbers compared to empty vector transduced cells (Supplementary Fig. S8).

## Discussion

Several prognostic gene expression signatures for AML patients have been reported, but were established based on *a priori* assumptions about the identity of the potentially relevant genes, and/or contained relatively large numbers of genes, making them too complex for potential clinical applications^7,12–20^. We therefore aimed to establish a signature composed of a small number of genes, both whose identity and number were determined through unbiased approaches. As a training set, a cohort of cytogenetically normal patients representing all age groups was used. The resulting signature, 4-GES (consisting of *SOCS2*, *IL2RA*, *NPDC1*, and *PHGDH*) was confirmed as an independent prognostic parameter in five of six additional data sets, including some that contained cytogenetically heterogeneous patient groups and/or employed different gene expression profiling technologies, and just fell short of significance in the sixth data set. When the 4-GES was applied to distinct cytogenetic subgroups of AML a highly significant prognostic value was observed for the cytogenetically intermediate subgroup, however, for the cytogenetically poor subgroup statistical significance was not reached after correction for multiple testing. Because the cytogenetically poor subgroup was rather small, the 4-GES should be evaluated in a much broader cohort of adverse cytogenetic risk AML patients in future studies. The 4-GES performed as well or better than other rigorously tested prognostic signatures, and was able to substantially refine the ELN classification both in younger (<60 years) and older (≥60 years) patients with AML. Further studies are necessary to develop a standardized, fast and easy to use (multiplex) real-time PCR approach for the 4 genes and housekeepers to stratify AML patients into low and high risk groups prospectively.

At present, risk stratification in AML is based on recurrent genetic alterations. In addition to their prognostic value, these contribute to various aspects of disease pathology, and some of them have been developed as therapeutic targets^2–6,37^. Some genes whose individual aberrant expression had prognostic significance in AML were also shown to contribute to leukemogenesis^9,10^, and in a recent report, a gene expression signature associated with LSCs and poor therapy response was used to identify targeted drugs potentially useful for the treatment of AML^8^. The 4-GES comprises only four genes, making it appear likely that most or all of these also make a functional contribution to AML pathogenesis. To begin to address this question, the role of *SOCS2* in AML was investigated using suitable experimental models. *SOCS2* was selected because it resulted as the top gene from the survival modeling approach; in addition, it was strongly and significantly up-regulated at relapse of AML, a disease stage that is often refractory to therapy and thus can be considered as inherently aggressive^38^. *SOCS* genes are transcriptional targets of activated JAK-STAT signalling^22^, a pathway that plays a major pro-leukemogenic role in AML, contributes to the growth and maintenance of AML LSCs^29^, and is being explored as target for rationally designed therapeutics^39^. SOCS proteins act as substrate recruiting components of E3-ubiquitin ligase complexes and initiate the degradation of cytokine receptors and signaling proteins, thus acting as negative feedback regulators of the cytokine activated pathways leading to their induction^22^. They would therefore be expected to serve as tumor suppressors, and *SOCS2* was indeed down-regulated in several cancer types^22^. In contrast, *SOCS2* was up-regulated in AML compared to healthy controls, and particularly in samples with activating ITD mutations of the tyrosine kinase receptor FLT3^40^. SOCS2 promoted the degradation of activated FLT3, and decreased proliferation and colony formation of cells experimentally expressing *FLT3*-ITD^40^; however, these experiments were performed using the murine pro-B cell line Ba/F3, which may not be an ideal model for human AML. In contrast, the data reported here show that experimental expression of *SOCS2* had a small but significant growth promoting effect in malignant human myeloid cell lines (Supplementary Fig. S8). Correspondingly, its knock-down in primary cells from the *Flt3*-ITD/*NPM1c* driven model of cytogenetically normal AML retarded proliferation and/or promoted cell death or senescence to an extent that precluded further experimentation (Supplementary Fig. S6B). A comparable, albeit less dramatic effect was observed with cells from an *MLL-AF9* driven AML model (Fig. 5C). In this model, knock-down of *Socs2* also decelerated leukemia onset in mice, promoted LC differentiation, and reduced the abundance, quiescence, and activity of LSCs (Fig. 5D–H), the LC subpopulation that acts as driver of the disease and is responsible for therapy resistance and relapse^1^. Together with the observation that high *SOCS2* expression is associated with a poor prognosis (Supplementary Fig. S1), these data provide strong support for an oncogenic role of this gene in AML. This may reflect a role of SOCS2 not only as a negative regulator of the JAK-STAT pathway, but also as a downstream target of it. Oncogenic roles of *SOCS2* were also reported in other tumor entities: SOCS2 levels were elevated in colon and prostate cancer, and high SOCS2 expression was associated with a poor prognosis in the latter^23,24^. The transcription of *SOCS2* was repressed by wild type p53, and it promoted proliferation, anchorage independent growth, resistance against basal and drug induced apoptosis, and tumor growth in xenograft assays in prostate and colon cancer cell lines^23–25^. Because our data regarding Socs2 expression and growth regulatory effects of Socs2 derived from *TP53* wt mice/cells and *TP53* mutant human myeloid cells, we suggest that these effects are independent of p53 and *TP53* mutations in AML. Other examples of a negative signalling regulator associated with poor prognosis include the dual-specificity phosphatase 6 (DUSP6) which regulates the basal levels of phosphorylated ERK in the cytosol^41,42^. High DUSP6 expression was found to be associated with poor survival in ALL and breast cancer patients^43,44^.

As for the other genes in the 4-GES, the *interleukin 2 receptor subunit alpha* (*IL2RA*, also named *CD25*) gene was specifically expressed on LSCs in a subset of patients with AML^45^, and was previously reported as an independent prognostic parameter able to further improve prognostication based on cytogenetic and mutational data^46^. An ongoing phase I study of patients with relapsed/refractory CD25-positive AML/ALL treated with the human monoclonal anti-CD25 antibody ADCT-301 (NCT02588092) showed an acceptable safety profile. Data about treatment response are not available yet^47^. However, an other trial (NCT02432235) tested efficacy of ADCT-301 in relapsed/refractory Hodgkin/Non-Hodgkin lymphoma patients and reported an overall response rate of 56%^48^.

The *neural proliferation, differentiation and control 1* (*NPDC1*) gene was cloned as a gene associated with contact inhibition of neuronal precursor cells^49^. It is one of the less intensely studied genes, but, interestingly, was significantly up-regulated at relapse of AML^38^.

Phosphoglycerate dehydrogenase (PHGDH) catalyzes the rate limiting step of serine synthesis from the glycolytic intermediate 3-phosphoglycerate; serine metabolism plays roles in both nucleotide biosynthesis and antioxidant defense. *PHGDH* is overexpressed in triple negative breast cancer, lung adenocarcinoma, and other tumors, and is associated with a poor prognosis. It contributes to tumor formation, stemness, metastasis, and chemotherapy resistance^50,51^, and is actively pursued as a therapeutic target^52,53^. Its role is far less well studied in leukemia, but serine deprivation and PHGDH inhibition were reported to cooperate to inhibit growth of the malignant human myeloid cell line HL60^54^.

In summary, we present here a gene expression signature (4-GES) that is a highly significant independent prognostic parameter throughout several independent AML cohorts. A major advantage over previously published signatures is that it is composed of only four genes, which makes its application in routine diagnostics feasible. Moreover, the 4-GES has the potential to refine the ELN classification, which is the current state of the art risk classification of AML. This refinement was associated with re-assignment of substantial numbers of patients into the prognostically poor subgroup. However, as outlined above, the genes constituting the 4-GES are likely to contribute to the pathological features of AML, raising the possibility that the 4-GES may not only be a prognosticator of poor outcome under standard therapy, but even represent a target for rationally designed therapies maybe improving outcome of the subgroup of AML that currently has the poorest prognosis.

## Materials and Methods

### Publicly available gene expression data sets

Four independent microarray data sets, 2 of which consist of 2 cohorts each, were obtained from the Gene Expression Omnibus (GEO) database, and The Cancer Genome Atlas (TCGA) RNA-seq data were downloaded from the Cancer Browser database (Table 1). For GSE37642, risk classification according to the ELN 2010 score was kindly provided by T. Herold, University of Munich, Department of Internal Medicine III, Munich, Germany. All samples with French American British (FAB) type M3, which mandates a substantially different treatment protocol than used for all other AML patients, and samples with unknown FAB type or myelodysplastic syndrome (MDS) were excluded. To optimize comparability among the microarray data sets, raw Affymetrix data were processed, normalised and log2 transformed using the frozen robust multiarray (fRMA) algorithm and R 3.4.2 software^55^. Pre-processed Illumina BeadArray data (GSE71014) and RNA-seq data (TCGA_LAML) were used as provided by the databases. Microarray data of DMSO/sorafenib treated Ba/F3-ITD cells and of human hematopoietic cells were obtained from ArrayExpress database (E-MTAB-4487, E-MEXP-1242) and GEO database (GSE42519, GSE17054, GSE19599, GSE11864). Heatmaps were generated on scaled log2 values using ClustVis^56^.

### Prognostic model selection and survival analyses

A prognostic gene expression signature was calculated by applying *rbsurv*, a robust likelihood-based survival modeling approach^57^, to cohort 1 of GSE12417. The samples in this data set were randomly distributed between a training and a validation set. Each gene was fitted to the training set and the parameter estimate for the association between expression of this gene and overall survival (OS) was calculated. With the parameter estimate, log likelihood to predict OS was evaluated in the validation set. This procedure was repeated 100 times, yielding 100 log-likelihoods per gene. The gene with the largest mean log likelihood (*SOCS2*) in the survival model was selected as the first top gene. By evaluating all possible two-gene models, the gene that together with *SOCS2* yielded the largest mean log likelihood was selected as the second top gene. This forward gene selection process was continued and resulted in a set of candidate genes for the prognostic model. Finally, an optimal prognostic model was selected based on the minimal Akaike information criterion (AIC) which takes the goodness of the model fit and the complexity (number of genes) into account.

For each patient in a data set, a score was calculated as the sum of the products of log2 transformed expression values of the model genes and their β-coefficients from a multivariable Cox regression, which was performed on cohort 1 of data set GSE12417 and included all genes. Optimal cut-offs for classification of patients into high-risk or low-risk groups were calculated for each data set using maximally selected rank statistics (R package *maxstat*). The Kaplan Meier method was used to estimate survival distributions and the log-rank test was used to evaluate statistical significance in OS between risk groups. P-values from these analyses were adjusted for multiple testing as described previously^58^. Uni- and multivariable Cox regressions were calculated using the *coxph* function in R. Univariate analyses were performed for all parameters provided along with the respective gene expression data (Supplementary Table S1). Only parameters which were significant in univariate analyses were included in multivariable analyses. A p-value of <0.05 was considered statistically significant. The R packages *survival* and *survminer* were used for these calculations and for data visualization. All statistical tests were performed using R 3.4.2.

### Ethics statement

Animal experiments were approved by the Animal Ethics Committee of the Medical University of Vienna and the Austrian Federal Ministry of Education, Science, and Research (GZ66.009/0309-WF/V/3b/2015). Federation of European Laboratory Animal Science Associations guidelines to minimize animal distress and suffering were followed.

### Isolation, culture, transduction, and transplantation of primary murine cells

The packaging cell lines Platinum-E (PlatE) and Phoenix-GP were maintained in DMEM (Invitrogen) supplemented with 10% fetal bovine serum (FBS) (Invitrogen) and 1% Penicillin/Streptomycin (Sigma-Aldrich). Primary murine hematopoietic or leukemic cells (LCs) were cultured in IMDM medium (Thermo Fisher Scientific) containing 10% FBS, 1% L-Glutamine (Thermo Fisher Scientific), 50 ng/ml mSCF, 10 ng/ml mIL-3, 10 ng/ml mTPO, 10 ng/ml mFlt3L (all from Peprotech), and 10 ng/ml mIL-6 (Biolegend). To generate mice with *MLL-AF9* driven AML, a retroviral transduction/transplantation approach was used. Lin^−^ Sca-1^+^ c-Kit^+^ (LSK) cells were isolated from bone marrow (BM) of 6–8 week old C57BL/6 mice (Department of Laboratory Animal Science & Genetics, Himberg, Austria). Retroviral particles were produced by calcium phosphate mediated co-transfection of PlatE cells with pMSCV_MLL-AF9_IRES_Venus^34^ (kindly provided by Dr. Johannes Zuber, Research Institute of Molecular Pathology, Vienna, Austria) and the ecotropic packaging plasmid psi2 (gag, pol, env). LSK cells (100,000 cells in 500 µl culture medium) were spinoculated with 1500 µl retroviral supernatant in the presence of 4 µg/ml polybrene (Sigma-Aldrich) and cytokines (as in the culture medium) for 1 h at 1300 rpm and 32 °C in a 12-well plate precoated with Retronectin (Takara). The infection was repeated with new retroviral supernatant after 5, 10, and 24 h, followed by a 48 h incubation in culture medium.

To knock down *Socs2*, two validated *Socs2* shRNAs (shSocs2 #361 and #362) and a non-target control shRNA (SHC012) in pLKO.1_puro_CMV_TagRFP (Mission© library, Sigma-Aldrich) were transfected into Phoenix-GP cells, along with the packaging plasmids pSPAX2 and pMDG.2, using calcium phosphate precipitation. Lentiviral particles were harvested after 48–72 h and used for spinoculation of spleen cells from mice with *MLL-AF9* or *FLT3*-ITD/*NPM1c*^36^ driven AML as described above. The infection was repeated with fresh lentiviral supernatant after 24 h. Three days later, fluorescence marker positive cells were sorted and used for *in vitro* assays and/or transplantation of recipient mice.

For transplantation, 6–8 week old female C57BL/6 recipient mice were sub-lethally irradiated (5 Gy), anaesthesized on the next day, and injected retro-orbitally with *MLL-AF9*-transduced LSK cells (160,000 cell/mouse; unsorted because of the strong selective advantage associated with *MLL-AF9* expression) or with shCtrl or shSocs2-transduced *MLL-AF9*^+^ LCs (300,000 Venus^+^ RFP^+^ cells/mouse). BM and spleen cells were harvested from terminally ill mice for use in downstream experiments.

### Flow cytometric assays to analyse LC differentiation and the abundance and quiescence of LSC enriched cells

To analyze the differentiation status of LCs, spleen cells from terminally ill mice were stained with fluorophor labelled antibodies against Gr-1 and Mac-1 (Supplementary Table S2) for 30 minutes on ice, washed once with staining buffer, and subjected to flow cytometry.

In the *MLL-AF9* AML model, LSCs are highly enriched in the Lin^−^ Sca1^−^ c-Kit^+^ CD34^+^ CD16/CD32^hi^ population^35,59^; we therefore defined Venus^+^ RFP^+^ cells with this immunophenotype as LSC enriched cells (LSCe). LSCe abundance among Venus^+^ RFP^+^ LCs was determined by flow cytometric analysis of leukemic spleen cells stained with the respective antibodies (Supplementary Table S2). To determine the proportion of quiescent LSCe, spleen cells from leukemic mice were stained with antibodies against LSCe surface markers, fixed and permeabilised in Cytofix/Cytoperm (BD Biosciences), stained with Ki-67 antibody (Supplementary Table S2) and DAPI (1 µg/mL; Sigma-Aldrich) in Perm/Wash™ Buffer (BD Biosciences), washed with Perm/Wash™ Buffer, and subjected to flow cytometry. Ki-67^−^ LSCe with a 2n DNA content were considered quiescent.

Flow cytometric analyses were performed on an LSR Fortessa SORP (BD Biosciences), and data were analysed with FlowJoX software (Treestar).

### Colony formation assay

BM cells from leukemic mice were sorted for Venus^+^ RFP^+^ positivity, and 2,000 cells from each genotype were seeded into methyl cellulose (MethoCult GF M3434; Stem Cell Technologies). Total colony formation was quantified after 7 days, and 2,000 cells per condition were used for replating.

### Quantitative RT-PCR

Total RNA was extracted using Trizol (Life Technologies) and reverse transcribed using random hexamer primers (Life Technologies) and M-MLV reverse transcriptase (Life Technologies). Quantitative RT-PCR (qRT-PCR) was performed on a Step One Plus Real Time PCR system (Life Technologies) using GoTaq qPCR Master Mix (Promega) and the following primers: *Socs2* (fwd: 5′-CTGCGCGAGCTCAGTCAAA-3′, rev: 5′-CAATCCGCAGGTTAGTCGGT-3′), *ß-2-microglobulin* (fwd: 5′_CCTTCAGCAAGGACTGGTCT-3′, rev: 5′-TGTCTCGATCCCAGTAGACG-3′). Assays were performed in triplicate, and *Socs2* expression was normalised to *ß-2-microglobulin* expression using the ΔΔC_T_ method^60^.

### Immunoblot analysis

Preparation of protein lysates, SDS-PAGE, transfer to PVDF membranes (Hybond-P; Amersham), and antibody incubations were performed using standard procedures. Blots were developed using SuperSignal West Femto or Pico Chemiluminescent Substrate (both from Thermo Scientific) and scanned using a ChemiDoc Touch Imaging System (Bio Rad). Densitometric analysis was performed with Image-J software (National Institutes of Health, Maryland, USA).

### Statistical analyses of experimental data

Differences between two independent groups were calculated using Student’s t-test, and differences between multiple groups were determined by 2-way ANOVA followed by Bonferroni’s post-hoc test. The log-rank test was used to evaluate survival differences between groups of mice. p-values < 0.05 were considered statistically significant. Analyses were performed using GraphPad Prism 6 software.

### Ethics approval

Animal experiments were approved by the Animal Ethics Committee of the Medical University of Vienna and the Austrian Federal Ministry of Education, Science, and Research (GZ66.009/0309-WF/V/3b/2015). Federation of European Laboratory Animal Science Associations guidelines to minimize animal distress and suffering were followed.

## Supplementary information

Supplementary Information
