# Supplementary material for: SOCS2 is part of a highly prognostic 4-gene signature in AML and promotes disease aggressiveness
Source: Sci Rep. 2019 Jun 24;9:9139. doi: 10.1038/s41598-019-45579-0 (PMC6591510; doi:10.1038/s41598-019-45579-0)
Supplement: Supplementary file 1 — Supplementary Information [file 41598_2019_45579_MOESM1_ESM.pdf]

**Supplementary information to the article "SOCS2 is part of a highly prognostic 4-gene signature in AML and promotes disease aggressiveness"**

Chi Huu Nguyen[1,2], Tobias Glüxam[1,2], Angela Schlerka[1,2], Katharina Bauer[1,2,3], Alexander M. Grandits[1,2], Hubert Hackl[4], Oliver Dovey[5], Sabine Zöchbauer-Müller[1,2], Jonathan L. Cooper[5], George S. Vassiliou[5], Dagmar Stoiber[6,7], Rotraud Wieser[1,2]* and Gerwin Heller[1,2,8]*

[1]Department of Medicine I, Division of Oncology, Medical University of Vienna, Vienna, Austria. [2]Comprehensive Cancer Center, Vienna, Austria. [3]Institute of Science and Technology Austria, Vienna, Austria, [4]Division of Bioinformatics, Biocenter, Medical University of Innsbruck, Innsbruck, Austria. [5]Wellcome Trust Sanger Institute, Wellcome Trust Genome Campus, Hinxton, UK. [6]Ludwig Boltzmann Institute for Cancer Research, Vienna, Austria. [7]Institute of Pharmacology, Center for Physiology and Pharmacology, Medical University of Vienna, Vienna, Austria. [8]Institute of Pharmacology and Toxicology, Department for Biomedical Sciences, University of Veterinary Medicine Vienna, Vienna, Austria.

**Supplementary Methods**

Cell culture, transduction of human myeloid cell lines, and proliferation assay

The packaging cell line Phoenix-GP was maintained in DMEM (Invitrogen) supplemented with 10% fetal bovine serum (FBS) (Invitrogen) and 1% Penicillin/Streptomycin (Sigma-Aldrich). U937 and HL60 cells and their derivatives were grown in RPMI 1640 (Invitrogen) containing 10% FBS and 1% Penicillin/Streptomycin/Glutamine (Life Technologies). A codon optimized version of the human *SOCS2* cDNA was cloned into the retroviral vector pMSCV_IRES_GFP using the *XhoI* and *EcoR*I restriction sites. The resulting vector, pMSCV_SOCS2_IRES_GFP, as well as empty pMSCV_IRES_GFP as a control, were transfected into Phoenix-GP cells, along with helper plasmids pMD2.G and pGag-Pol, and retroviral particles were harvested and used to infect U937 and HL60 cells through standard procedures. Three days later, the resulting cell lines U937_Vec, U937_SOCS2, HL60_Vec, and HL60_SOCS2 were sorted for GFP positivity on an Astrios (Beckman Coulter). For the proliferation assays, cells were seeded into 12-well plates at a density of $1.5x10^5$ cells/ml, split at equal ratios when necessary, and total viable cells were counted every 24 h using a CASY counter (Roche Innovates).

## Supplementary Figures

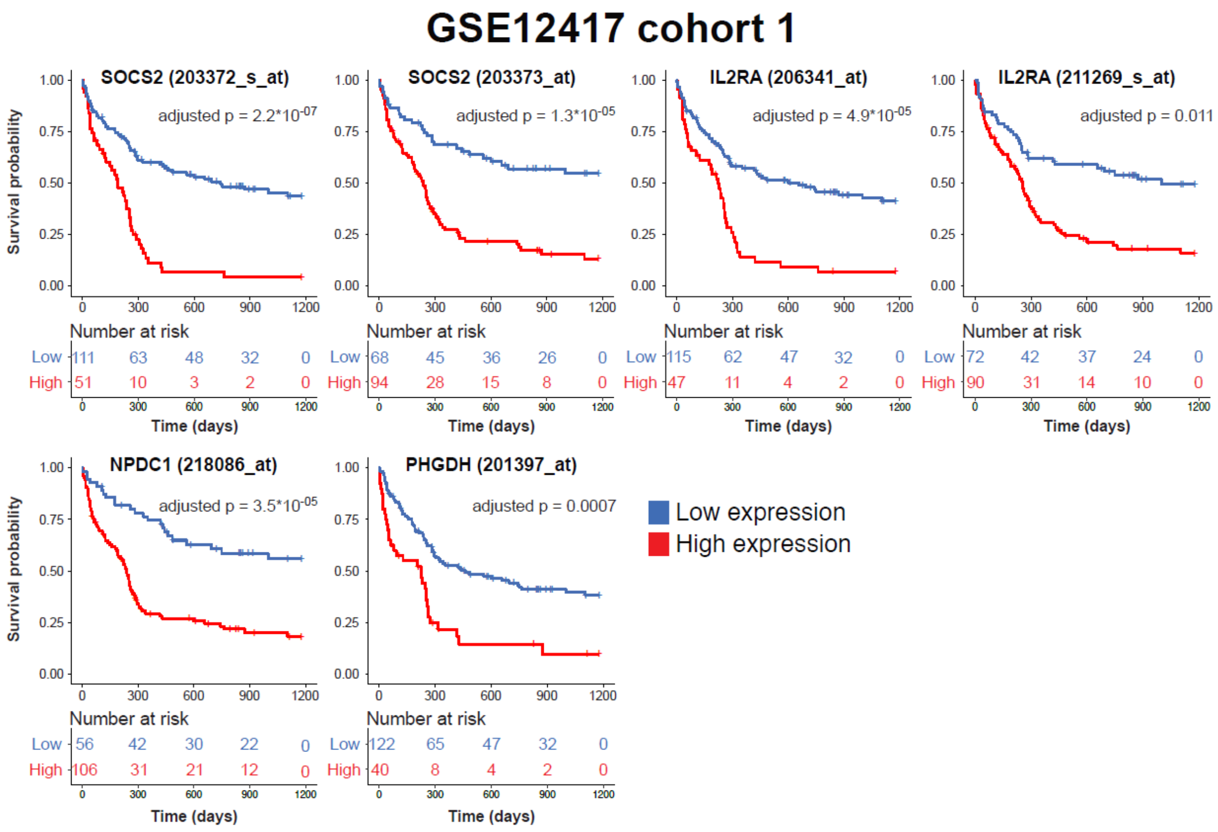


**Supplementary Figure S1.** Kaplan Meier curves for overall survival of 162 cytogenetically normal AML patients (GSE12417, cohort 1) according to expression values for alternative probe sets for *SOCS2*, *IL2RA*, *NPDC1* and *PHGDH*. Expression values were dichotomized by maximally selected rank statistics. Statistical significance was calculated using the log rank test and p-values were adjusted for multiple testing as described by Altman *et al.* [1].

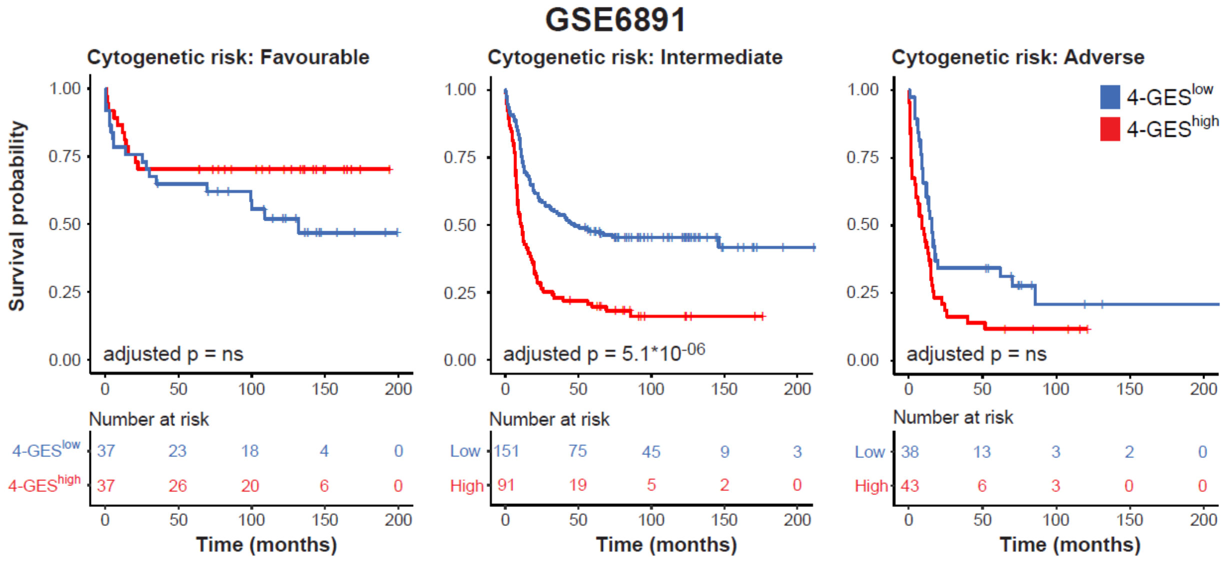


**Supplementary Figure S2.** Kaplan Meier curves for overall survival of AML patients classified as cytogenetically favourable, intermediate, or adverse (GSE6891, combined cohorts 1 and 2). Patients were stratified into 4-GES$^{low}$ (blue) and 4-GES$^{high}$ (red) subgroups. Statistical significance was calculated using the log rank test and p-values were adjusted for multiple testing as described by Altman *et al.* [1].

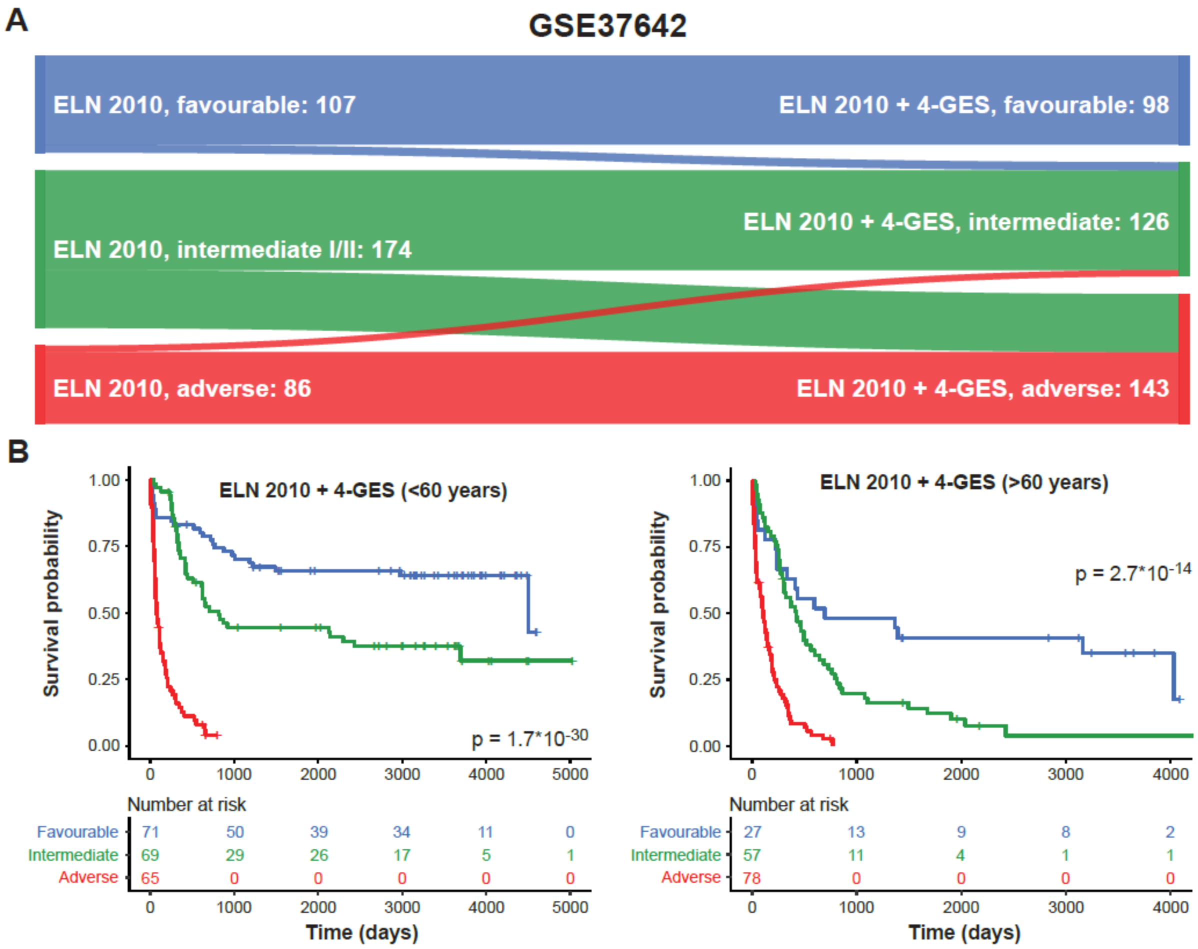


**Supplementary Figure S3.** The 4-GES is able to refine the ELN classification of AML patients included in the GSE37642 data set. **(A)** A shift of patients from ELN 2010 favourable, intermediate I/II and adverse (left side) to ELN 2010 + 4-GES favourable, intermediate and adverse (right side) is shown. ELN favourable/4-GES$^{high}$ patients with low median OS were re-assigned to ELN intermediate risk group, ELN intermediate/4-GES$^{high}$ patients with low median OS were re-assigned to ELN adverse risk group, and ELN adverse/4-GES$^{low}$ patients with high median OS were re-assigned to ELN intermediate risk group. **(B)** Kaplan Meier curves for overall survival of AML patients <60 years of age (left) and >60years of age (right) stratified into favourable, intermediate, and adverse risk based on the ELN 2010 + 4-GES classification. ELN, European Leukemia Net.

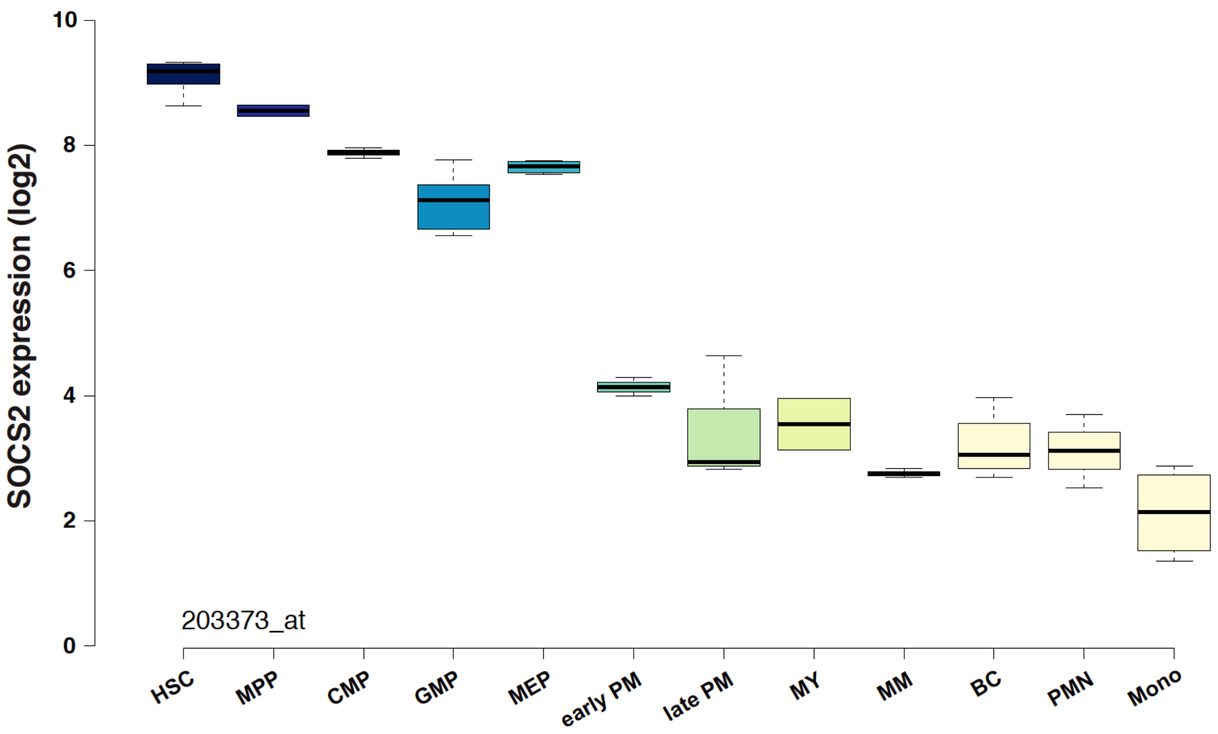


**Supplementary Figure S4.** SOCS2 (Affymetrix probe set: 203373_at) expression in various hematopoietic cells determined by gene expression microarray analyses. Log2 transformed gene expression values are shown. HSC, Hematopoietic stem cell; MPP, Multipotential progenitors; CMP, Common myeloid progenitor cell; GMP, Granulocyte monocyte progenitors; MEP, Megakaryocyte-erythroid progenitor cell; early_PM, Early Promyelocyte; late_PM, Late Promyelocyte; MY, Myelocyte; MM, Metamyelocytes; BC, Band cell; PMN, Polymorphonuclear cells; Mono, Monocytes

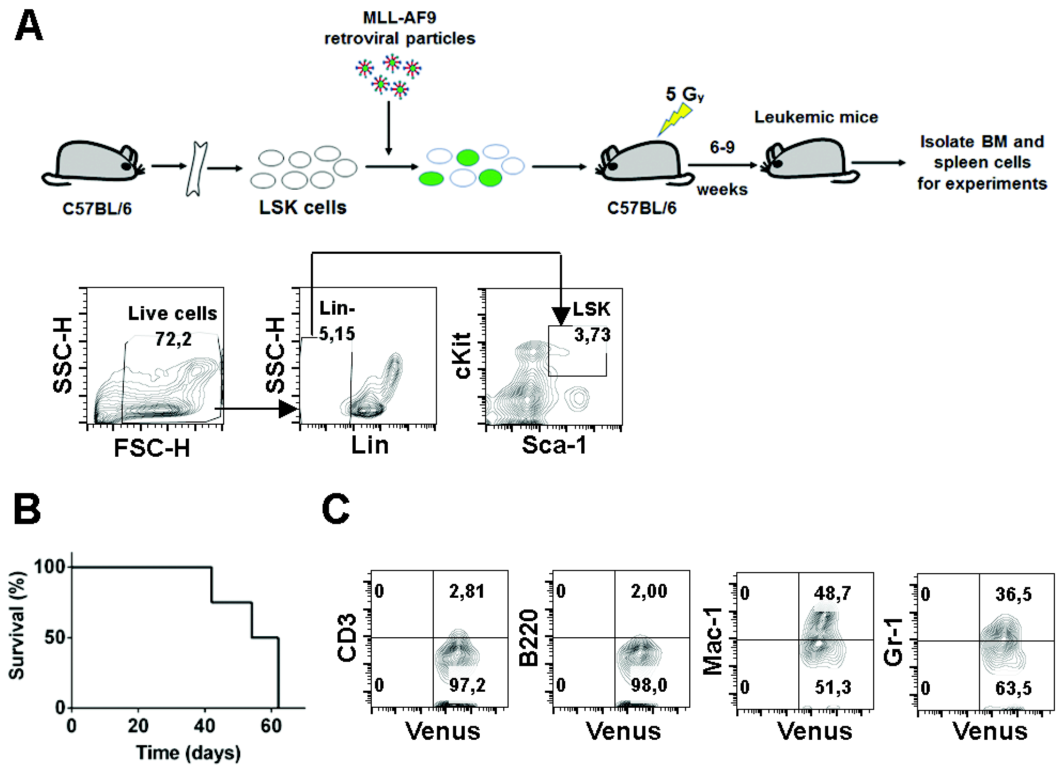


**Supplementary Figure S5.** *MLL-AF9*-transduced LSK cells cause an aggressive AML-like disease in mice. **(A)** Schematic overview of the experiment. **(B)** Kaplan Meier plot of mice transplanted with *MLL-AF9*-transduced LSK cells (160,000 unsorted cells per mouse, n = 3). (**C**) Flow cytometric analysis of spleen cells from terminally ill mice confirmed the expression of myeloid (Mac-1, Gr-1) and the absence of lymphoid (CD3, B220) markers in LCs (Venus$^{+}$ cells; representative experiment).

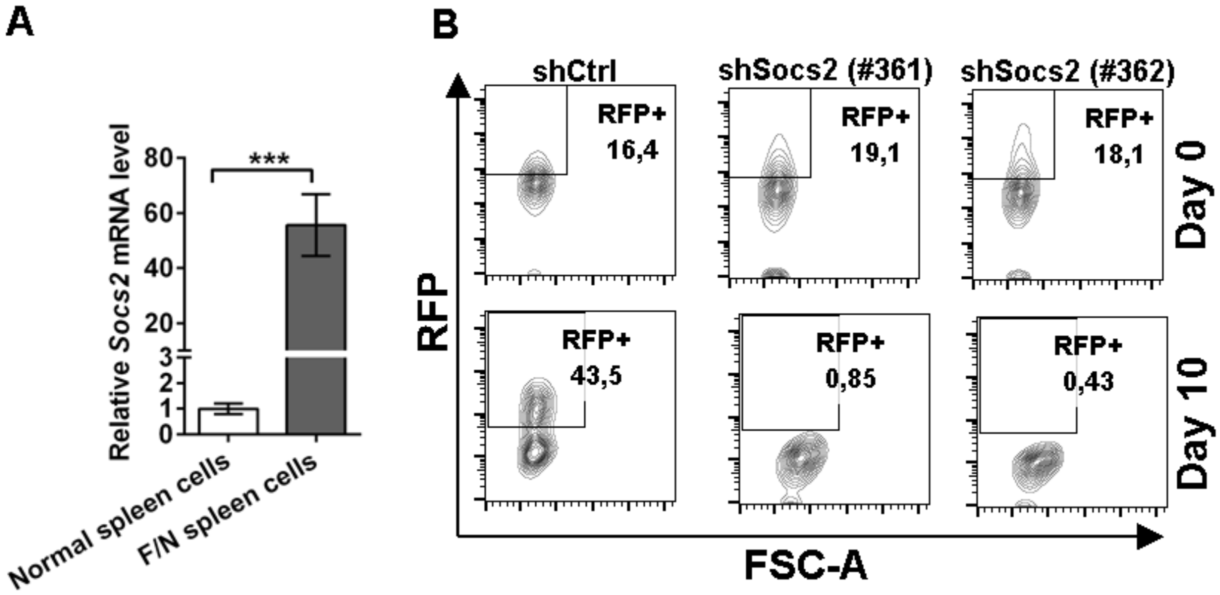


**Supplementary Figure S6.** *Socs2* is required for proliferation and/or survival of *Flt3*-ITD/*NPM1c* LCs *in vitro*. **(A)** *Socs2* mRNA levels in spleen cells from *Flt3*-ITD/*NPM1c* (F/N) leukemic mice and from healthy mice were determined by qRT-PCR and normalised to those of the housekeeping gene *ß-2-microglobulin* using the $\Delta\Delta C_T$ method. Mean ± SEM of three independent experiments; ***, $p < 0.001$ (Student's two-tailed t-test). **(B)** Representative flow cytometric analysis of $RFP^+$ cells from shCtrl or shSocs2 transduced *Flt3*-ITD/*NPM1c* LCs. Upper panel, cells on the day of sorting (day 0); lower panel, sorted cells after 10 days in suspension culture (day 10).

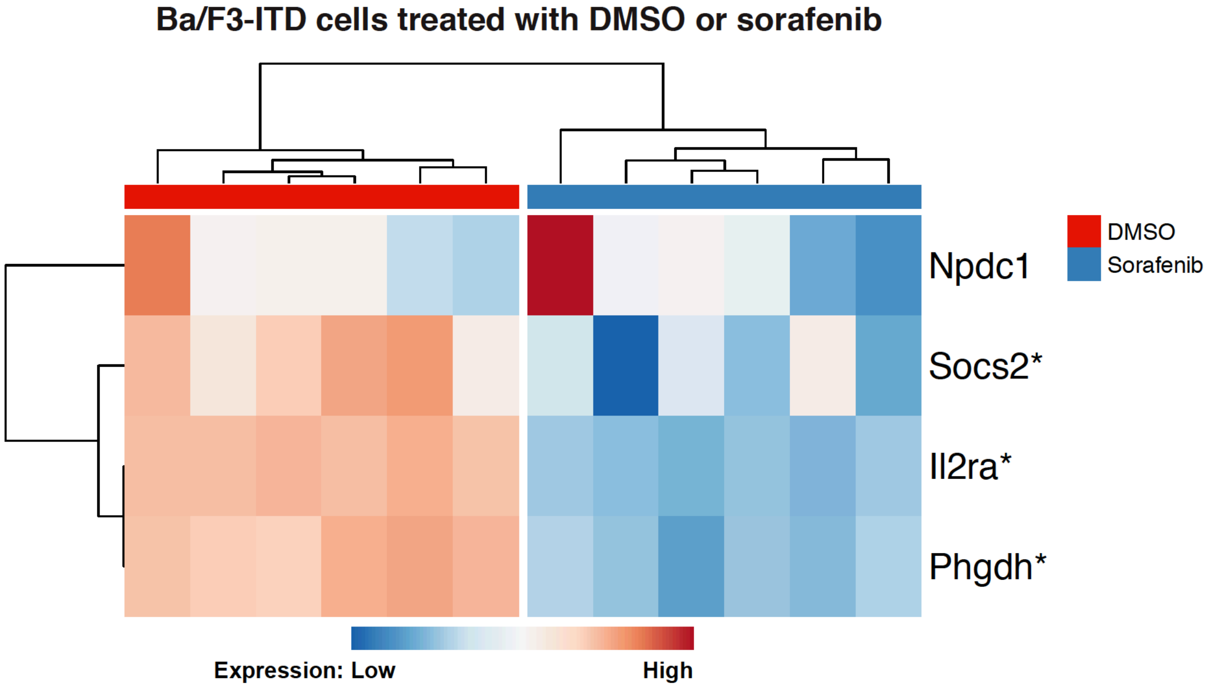


**Supplementary Figure S7.** Expression of *Npdc1*, *Socs2*, *Il2ra* and *Phgdh* in DMSO (n = 6) or sorafenib (n = 6) treated Ba/F3-ITD cells determined using MoGene-1_0-st-v1 microarrays (ArrayExpress ID: E-MTAB-4487). Ba/F3-ITD cells were treated with DMSO or 10nM sorafenib for 24 hours. Log2 transformed gene expression values are shown (blue, low expression; red, high expression). Asterisks indicate statistically significant differences in gene expression between DMSO and sorafenib treated cells (FDR < 0.005).

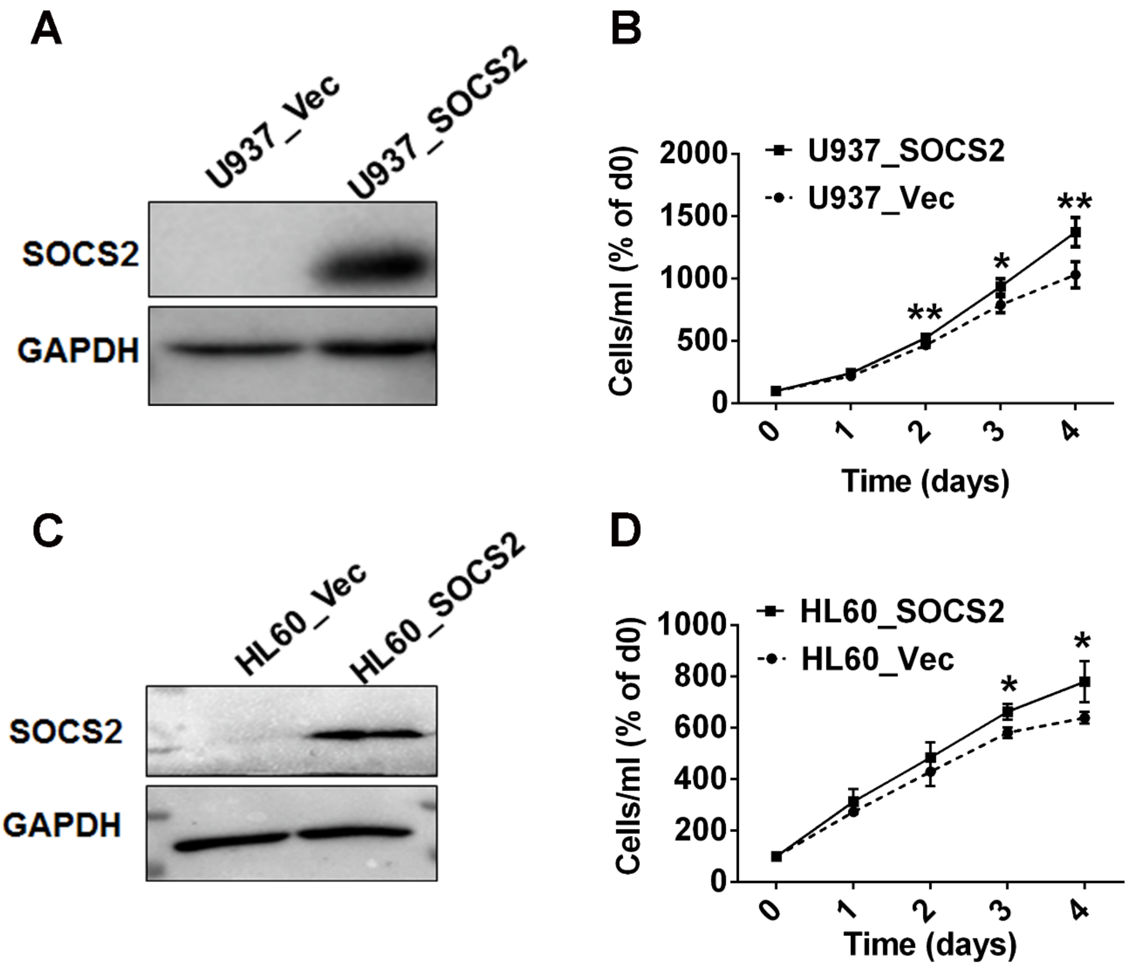


**Supplementary Figure S8.** Experimental expression of *SOCS2* promotes proliferation of the malignant human myeloid cell lines U937 **(A, B)** and HL60 **(C, D)**. **(A, C)** Immunoblot analyses demonstrating expression of SOCS2 in U937_SOCS2 and HL60_SOCS2, but not U937_Vec and HL60_Vec cells. **(B, D)** Relative counts of U937_SOCS2, U937_Vec, HL60_SOCS2, and HL60_Vec cells over a period of 4 days. Mean ± SEM of three independent experiments. *, $p < 0.05$; **, $p < 0.01$ (2-way ANOVA followed by Bonferroni's post-hoc test).

**Supplementary Table S1.** Univariable Cox regression analysis for overall survival of AML patients

| **Data set** | **Variable** | **HR** | **95% CI** | **p-value** |
|---|---|---|---|---|
| **GSE12417 cohort 1** | 4-GES$^{high}$ vs. 4-GES$^{low}$ | 4.09 | 2.52-6.64 | **1.3*10$^{-08}$** |
| | Age (years) | 1.03 | 0.76-0.97 | **0.0005** |
| | FAB, M1 vs. others | 1.44 | 0.51-4.07 | 0.491 |
| | FAB, M2 vs. others | 1.1 | 0.39-3.12 | 0.858 |
| | FAB, M4 vs. others | 0.71 | 0.24-2.07 | 0.533 |
| | FAB, M5 vs. others | 0.82 | 0.26-2.58 | 0.735 |
| | FAB, M6 vs. others | 0.61 | 0.14-2.72 | 0.516 |
| **GSE12417 cohort 2**[a] | 4-GES$^{high}$ vs. 4-GES$^{low}$ | 4.93 | 1.19-20.35 | **0.027** |
| | Age (years) | 1.04 | 1.01-1.06 | **0.006** |
| **GSE6891 cohort 1** | 4-GES$^{high}$ vs. 4-GES$^{low}$ | 2.33 | 1.68-3.25 | **4.5*10$^{-07}$** |
| | Age (years) | 1.01 | 0.99-1.03 | 0.066 |
| | FAB, M1 vs. others | 0.5 | 0.2-1.28 | 0.149 |
| | FAB, M2 vs. others | 0.46 | 0.18-1.17 | 0.103 |
| | FAB, M4 vs. others | 0.48 | 0.18-1.24 | 0.127 |
| | FAB, M5 vs. others | 0.68 | 0.27-1.72 | 0.419 |
| | FAB, M6 vs. others | 0.16 | 0.02-1.41 | 0.1 |
| | Cytogenetic risk[b] | 1.96 | 1.55-2.46 | **1.2*10$^{-08}$** |
| | Sex | 1.22 | 0.89-1.68 | 0.226 |
| | *FLT3*-ITD | 1.82 | 1.29-2.56 | **0.0007** |
| | *FLT3*-TKD | 0.72 | 0.42-1.25 | 0.248 |
| | *IDH1* | 0.66 | 0.32-1.35 | 0.252 |
| | *IDH2* | 1.15 | 0.65-2.04 | 0.627 |
| | *KRAS* | 0.92 | 0.23-3.7 | 0.903 |
| | *NRAS* | 0.63 | 0.36-1.12 | 0.116 |
| | *NPM1c* | 0.81 | 0.57-1.15 | 0.241 |
| | *CEBPA* (wt/mono/bi) | 0.67 | 0.46-0.98 | **0.04** |
| | *CEBPA* (yes/no) | 0.46 | 0.2-0.97 | **0.042** |
| | *EVI1* expression | 1.81 | 1.09-3.01 | **0.022** |
| **GSE6891 cohort 2** | 4-GES$^{high}$ vs. 4-GES$^{low}$ | 2.48 | 1.7-3.62 | **2.5*10$^{-06}$** |
| | Age (years) | 1.02 | 1-1.03 | **0.017** |

| | | | | |
|---|---|---|---|---|
| | FAB, M1 vs. others | 0.67 | 0.3-1.48 | 0.319 |
| | FAB, M2 vs. others | 0.7 | 0.32-1.53 | 0.368 |
| | FAB, M4 vs. others | 0.64 | 0.29-1.44 | 0.285 |
| | FAB, M5 vs. others | 0.67 | 0.3-1.49 | 0.325 |
| | FAB, M6 vs. others | 0.78 | 0.17-3.69 | 0.755 |
| | Cytogenetic risk[b] | 1.57 | 1.14-2.16 | **0.005** |
| | Sex | 0.91 | 0.63-1.33 | 0.64 |
| | *FLT3*-ITD | 1.44 | 0.97-2.15 | 0.072 |
| | *FLT3*-TKD | 0.67 | 0.33-1.38 | 0.275 |
| | *IDH1* | 1.15 | 0.6-2.2 | 0.672 |
| | *IDH2* | 0.59 | 0.29-1.22 | 0.158 |
| | *NRAS* | 0.98 | 0.54-1.78 | 0.942 |
| | *NPM1c* | 0.96 | 0.64-1.44 | 0.86 |
| | *CEBPA* (wt/mono/bi) | 0.76 | 0.47-1.22 | 0.255 |
| | *CEBPA* (yes/no) | 0.68 | 0.3-1.55 | 0.361 |
| | *EVI1* expression | 3.52 | 1.85-6.69 | **0.0001** |
| **GSE37642** | 4-GES$^{high}$ vs. 4-GES$^{low}$ | 2.65 | 2.04-3.45 | **$3.1*10^{-13}$** |
| | Age (years) | 1.04 | 1.03-1.05 | **$2.9*10^{-14}$** |
| | FAB, M2 vs. others | 0.75 | 0.54-1.03 | 0.077 |
| | FAB, M4 vs. others | 0.51 | 0.36-0.72 | **0.0001** |
| | FAB, M5 vs. others | 0.92 | 0.62-1.36 | 0.669 |
| | FAB, M6 vs. others | 0.78 | 0.41-1.48 | 0.449 |
| | FAB, M7 vs. others | 1.92 | 0.47-7.86 | 0.366 |
| | ELN score[c] | 1.43 | 0.7-1.28 | **$2.3*10^{-11}$** |
| **TCGA_LAML** | 4-GES$^{high}$ vs. 4-GES$^{low}$ | 1.91 | 1.28-2.86 | **0.0016** |
| | Age (years) | 1.04 | 0.97-1.02 | **$1.1*10^{-05}$** |
| | FAB, M1 vs. others | 1.14 | 0.54-2.39 | 0.737 |
| | FAB, M2 vs. others | 1.12 | 0.53-2.37 | 0.766 |
| | FAB, M4 vs. others | 1.14 | 0.55-2.28 | 0.72 |
| | FAB, M5 vs. others | 1.56 | 0.67-3.62 | 0.303 |
| | FAB, M6 vs. others | 2.71 | 0.59-12.55 | 0.202 |
| | FAB, M7 vs. others | 2.61 | 0.71-9.59 | 0.149 |
| | Cytogenetic risk[a] | 1.55 | 1.08-2.23 | **0.017** |

| | | | | |
|---|---|---|---|---|
| | Sex | 1 | 0.67-1.5 | 0.97 |
| | *FLT3*-ITD | 0.97 | 0.49-1.93 | 0.934 |
| | *FLT3*-TKD | 1.47 | 0.71-3.04 | 0.3 |
| | *IDH1* | 0.69 | 0.4-1.18 | 0.178 |
| | *RAS* | 0.78 | 0.31-1.92 | 0.584 |
| | *NPM1c* | 1.01 | 0.65-1.57 | 0.955 |

4-GES, 4-gene expression score; HR, hazard ratio; CI, confidence interval; FAB, French American British classification; [a], FAB classification of GSE12417 cohort 2 was excluded from univariable analyses because of low subgroup sample numbers; *FLT3*-ITD, *FLT3* internal tandem duplication; *FLT3*-TKD, *FLT3* tyrosine kinase domain mutation; gene names in italics refer to mutations of the respective genes; mono, monoallelic; bi, biallelic. [b], Assignment to cytogenetic risk groups were included in the respective GEO entries. [c], Assignment to ELN risk groups was provided by T. Herold, University of Munich, Department of Internal Medicine III, Munich, Germany. No relevant patient data were provided in GSE71014; therefore, regression analyses were not performed.

**Supplementary Table S2.** Antibodies used for immunoblot analysis and flow cytometry

| **Method** | **Target** | **Clone/source organism** | **Fluorophor/ conjugate** | **Company** | **Cat. no** | **Dilution** |
|---|---|---|---|---|---|---|
| IB | Human/mouse SOCS2 | Rabbit | - | Cell Signaling | 2779S | 1:1,000 |
| | Human/mouse GAPDH | 14C10 | - | Cell Signaling | 2118S | 1:50,000 |
| | Rabbit IgG | Goat | Horseradish peroxidase | Jackson ImmunoResearch | 111035008 | 1:5,000 |
| FC | Mouse Gr-1 | RB6-8C5 | AF700 | Biolegend | 108422 | 1:100 |
| | MouseMac-1 | M1/70 | AF700 | Biolegend | 101222 | 1:100 |
| | Mouse CD3 | 17A2 | AF700 | Biolegend | 100216 | 1:100 |
| | Mouse B220 | RA3-6B22 | AF700 | Biolegend | 103232 | 1:100 |
| | Mouse Ter119 | TER119 | AF700 | Biolegend | 116220 | 1:100 |
| | Mouse c-Kit | 2B8 | PE | Biolegend | 105808 | 1:50 |
| | Mouse c-Kit | 2B8 | APC-Cy7 | Biolegend | 105826 | 1:50 |
| | Mouse Sca-1 | D7 | BV421 | BD Bioscience | 562729 | 1:50 |
| | Mouse Sca-1 | D7 | PerCP/Cy5.5 | Biolegend | 122524 | 1:50 |
| | Mouse CD34 | RAM34 | FITC | eBioscience | 11034182 | 1:50 |
| | Mouse CD34 | MEC14.7 | PE/Cy5.5 | Biolegend | 119312 | 1:50 |
| | Mouse CD16/CD32 | 93 | PE/Cy7 | eBioscience | 25016182 | 1:50 |
| | Mouse Ki-67 | 16A8 | APC | Biolegend | 652406 | 1:50 |

IB, immunoblot analysis; FC, flow cytometry. Gr-1, Mac-1, CD3, B220, and Ter119 antibodies were combined to define $Lin^-$ cells.

**Supplementary Table S3.** Genes included in the 4-GES and in 3 published gene expression signatures

| **4-GES** | **L-24** | **M-7** | **W-3** |
|---|---|---|---|
| *IL2RA* | *ALS2CR8* | *CD34* | *CAP1* |
| *NPDC1* | *ANGEL1* | *F2RL1* | *CXCR6* |
| *PHGDH* | *ARL6IP5* | *FAM92A1* | *FAM124B* |
| *SOCS2* | *BSPRY* | *MIR155HG* | |
| | *BTBD3* | *RHOC* | |
| | *C1RL* | *SCRN1* | |
| | *CPT1A* | *VWA8* | |
| | *DAPK1* | | |
| | *ETFB* | | |
| | *FGFR1* | | |
| | *HEATR6* | | |
| | *LAPTM4B* | | |
| | *MAP7* | | |
| | *NDFIP1* | | |
| | *PBX3* | | |
| | *PLA2G4A* | | |
| | *PLOD3* | | |
| | *PTP4A3* | | |
| | *SLC25A12* | | |
| | *SLC2A5* | | |
| | *TMEM159* | | |
| | *TRIM44* | | |
| | *TRPS1* | | |
| | *VAV3* | | |

L-24, 24-gene expression signature by Li et al. [2]; M-7, 7-gene expression signature by Marcucci et al. [3]; W-3, 3-gene expression signature by Wilop et al. [4]

**Supplementary Table S4.** Univariable Cox regression analysis for overall survival of AML patients with respect to the 4-GES and 3 published gene expression signatures

| | **4-GES** | | **L-24** | | **M-7** | | **W-3** | |
|---|---|---|---|---|---|---|---|---|
| **Data set** | **HR** | **p** | **HR** | **p** | **HR** | **p** | **HR** | **p** |
| **GSE12417, cohort 1** | **4.09** | **$1.3*10^{-08}$** | **2.19** | **0.00012** | na | na | **1.47** | **0.03** |
| **GSE12417, cohort 2** | **4.93** | **0.027** | 1.63 | 0.097 | **2.68** | **0.001** | **1.97** | **0.016** |
| **GSE37642** | **2.62** | **$1.2*10^{-13}$** | **1.59** | **0.00011** | na | na | 1.15 | 0.171 |
| **GSE6891, cohort1** | **2.34** | **$4.5*10^{-07}$** | **1.71** | **0.001** | **1.95** | **$6.3*10^{-05}$** | 1.34 | 0.082 |
| **GSE6891, cohort2** | **2.48** | **$2.4*10^{-06}$** | **1.69** | **0.007** | **1.63** | **0.011** | 1.26 | 0.249 |
| **GSE71014** | **3.28** | **0.002** | 1.74 | 0.102 | **2.7** | **0.005** | 1.37 | 0.418 |
| **TCGA_LAML** | **1.91** | **0.002** | **1.78** | **0.006** | 1.16 | 0.471 | **2.19** | **0.01** |

L-24, 24-gene expression signature by Li et al. [2]; M-7, 7-gene expression signature by Marcucci et al. [3]; W-3, 3-gene expression signature by Wilop et al. [4]; HR, hazard ratio; na, not applicable (2 signature genes not represented on U133A arrays). Significant p-values and corresponding HRs are indicated in bold.

**Addendum to main Figure 5B: full length blot image**

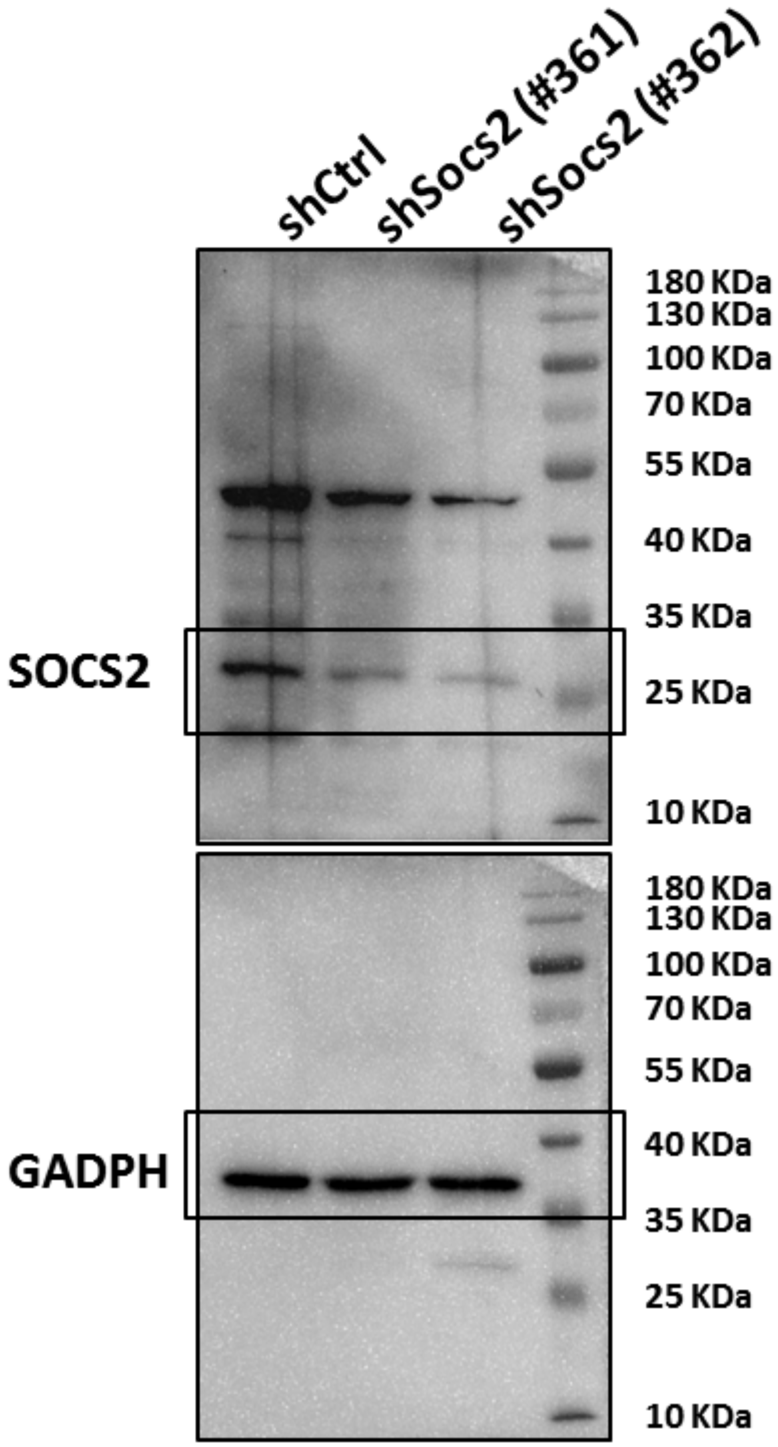

**Addendum to supplementary Figures S8A and S8C: full length blot images**

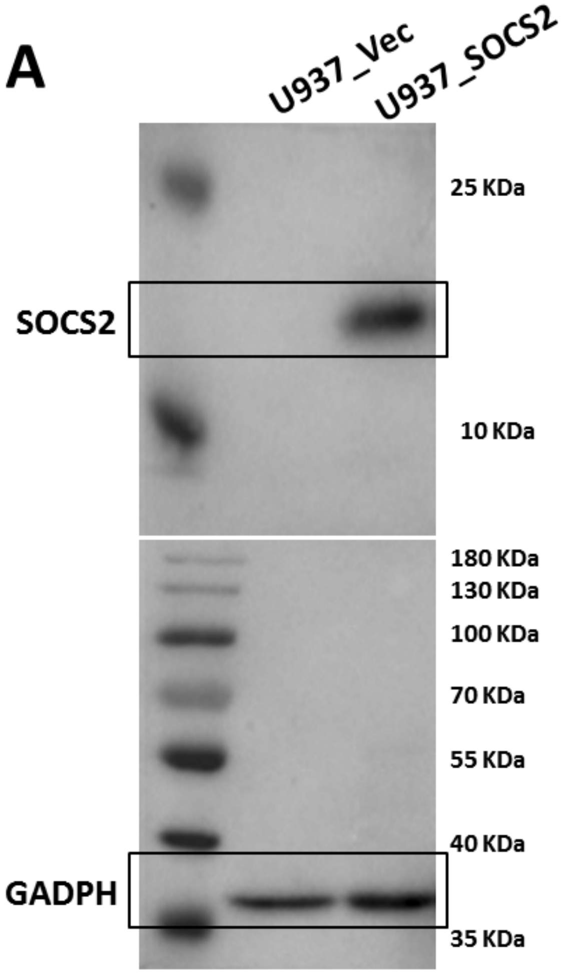


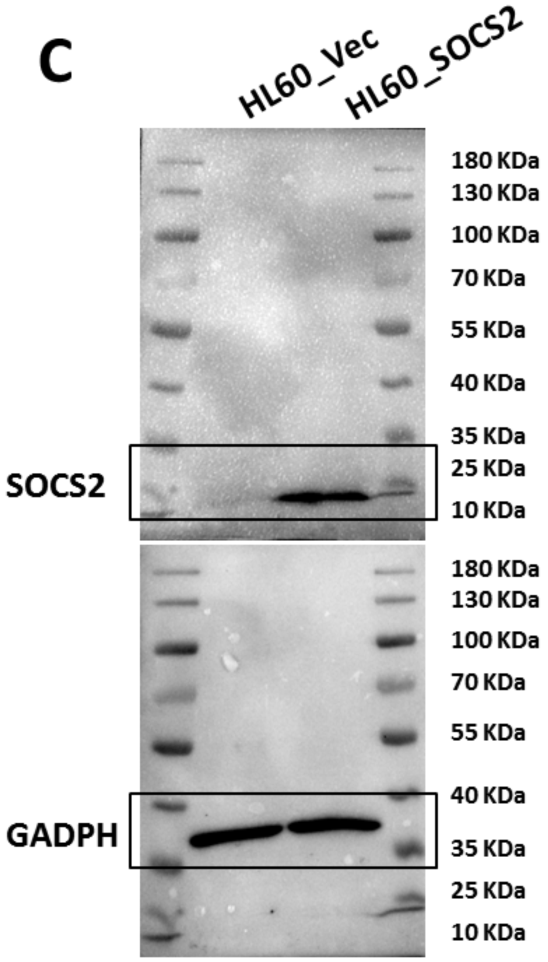

**Supplementary References**

1 Altman, D. G., Lausen, B., Sauerbrei, W. & Schumacher, M. Dangers of using "optimal" cutpoints in the evaluation of prognostic factors. *J Natl Cancer Inst* **86**, 829-835 (1994).

2 Li, Z. *et al.* Identification of a 24-gene prognostic signature that improves the European LeukemiaNet risk classification of acute myeloid leukemia: an international collaborative study. *J Clin Oncol* **31**, 1172-1181, doi:10.1200/JCO.2012.44.3184 (2013).

3 Marcucci, G. *et al.* Epigenetics meets genetics in acute myeloid leukemia: clinical impact of a novel seven-gene score. *J Clin Oncol* **32**, 548-556, doi:10.1200/JCO.2013.50.6337 (2014).

4 Wilop, S. *et al.* A three-gene expression-based risk score can refine the European LeukemiaNet AML classification. *J Hematol Oncol* **9**, 78, doi:10.1186/s13045-016-0308-8 (2016).
